# Metagenomic Insights into the Abundance of Iron-Reducing Microorganisms in a Petroleum-Contaminated Iron-Rich Aquifer

**DOI:** 10.3390/microorganisms13020433

**Published:** 2025-02-17

**Authors:** He Di, Min Zhang, Zhuo Ning, Changli Liu, Ze He, Shuaiwei Wang, Siyu Kong, Shuang Gan, Weichao Sun, Zhe Xu, Jinjin Ti

**Affiliations:** 1 Institute of Hydrogeology and Environmental Geology, Chinese Academy of Geological Sciences, Shijiazhuang 050061, China; 3020190008@email.cugb.edu.cn (H.D.); ningzhuozhuo@163.com (Z.N.); liuchangli@vip.163.com (C.L.); heze25@163.com (Z.H.); tairan_w@163.com (S.W.); 13273188379@163.com (S.K.); ganshuang2016@163.com (S.G.); 17730568088@163.com (W.S.); xu991102021@163.com (Z.X.); tijinjin22@mails.ucas.ac.cn (J.T.); 2 School of Chinese Academy of Geological Sciences, China University of Geosciences (Beijing), Beijing 100086, China; 3 Key Laboratory of Groundwater Remediation of Hebei Province & China Geological Survey, Zhengding 050083, China; 4 Key Laboratory of Water Cycle and Ecological Geological Processes, Xiamen 361021, China

**Keywords:** petroleum pollution, iron-reducing microorganisms (IRMs), metagenomics, hydrocarbon degradation, environmental remediation

## Abstract

In petroleum-contaminated aquifers, iron (III) serves as an electron acceptor, enabling microbial degradation of organic matter. While previous studies have focused on iron reduction and organic matter degradation under laboratory conditions, research on iron-associated microorganisms in petroleum-contaminated aquifers is limited. To explore the diversity and distribution of such microorganisms in natural settings, this study used metagenomic analysis of an iron-rich, petroleum-contaminated aquifer. Sixteen groundwater samples from both pollution source and background areas were collected for species annotation and functional gene identification. Results show more than 7000 species were identified as iron-reducing microorganisms (IRMs), including several previously well-characterized iron-reducing species (e.g., *Geobacter luticola* and *Geobacter sulfurreducens*). However, the majority of IRMs were not found in existing iron-reducing microbial databases. Some of them, such as *Sulfurospirillum* sp. and *Extensimonas perlucida*, could be taxonomically classified at the species level, while most were only annotated as unclassified bacteria. In the contamination source zone, these microorganisms proliferated extensively, which led to hydrocarbon degradation predominantly driven by iron reduction in the aquifer. This study enhances our understanding of hydrocarbon-degrading microorganisms and supports the management of petroleum-contaminated sites.

## 1. Introduction

Petroleum contamination in aquifers presents a significant environmental challenge, with hydrocarbon degradation largely dependent on microbial-mediated redox processes. In iron-rich environments, ferric iron (Fe(III)) serves as a critical electron acceptor, facilitating the anaerobic degradation of hydrocarbons by iron-reducing microorganisms (IRMs) [1,2]. These microorganisms play a crucial role in biogeochemical cycling by coupling iron reduction with the breakdown of complex organic pollutants. Through enzymatic pathways and electron transport mechanisms, IRMs convert Fe(III) to ferrous iron (Fe(II)), thereby driving contaminant degradation and influencing the redox dynamics of subsurface environments.

To date, IRMs have been identified and isolated under laboratory approaches (see Supplementary Table S1 List of IRMs) [3,4,5,6]. For example, microbial strains have been enriched from oil-contaminated sediments and groundwater using aromatic hydrocarbons as substrates. These include species from genera, such as *Geobacter*, *Thermincola*, and *Desulfuromonas*, and families, such as Peptococcaceae and Desulfobulbaceae [7,8,9,10,11,12]. Notable studies have demonstrated the metabolic potential of these microorganisms in anaerobic hydrocarbon degradation coupled to ferric iron [Fe(III)] reduction, with enriched microbial communities dominated by *Geobacteraceae*, *Carnobacteriaceae*, and *Anaerolinaceae* in petroleum-contaminated soils and mixed sludge systems [13]. The addition, iron and sulfate has been shown to enhance aromatic hydrocarbon degradation, with iron-reducing taxa, such as the genus *Geobacter* and the family Thermodesulfovibrionaceae playing critical roles [14].

Few studies have directly identified IRMs microbial communities in situ. For instance, Bekins et al. delineated redox zones in crude oil-contaminated aquifers and identified spatial distributions of IRMs, but without detailed microbial community analysis [15]. Sheng et al. (2016) reported a significant correlation between microbial community composition and hydrocarbon contamination levels. The relative abundance of key petroleum-degrading microorganisms, such as *Pseudomonas* and *Bacillus*, was positively associated with contamination gradients, and iron availability was identified as a key driver of microbial community assembly [16]. Similarly, Castro et al. (2022) demonstrated that the metabolic activity of IRMs, such as *Geobacter*, was strongly linked to the concentration of electron acceptors, such as Fe(III) [5]. Additionally, Ning et al. (2018) observed a positive correlation between the abundance of iron-reducing bacteria and the concentrations of petroleum hydrocarbons and Fe^2+^ [17].

Although previous studies have highlighted positive correlations between iron-reducing bacteria, hydrocarbon concentration, and iron levels, the underlying metabolic microorganisms, as well as their pathways and functional interactions, remain poorly understood. This knowledge gap limits our understanding of the ecological roles of IRMs in contaminated subsurface environments and their adaptive responses to geochemical conditions. Comprehensive insights into their ecological functions are crucial for elucidating their roles in hydrocarbon degradation and for advancing environmental remediation technologies.

This study investigates IRMs in petroleum-contaminated aquifers using metagenomic approaches integrated with hydrochemical profiling. By systematically characterizing the taxonomic diversity and functional potential of Fe(III)-reducing microbial populations in an iron-rich aquifer, the research aims to elucidate their metabolic pathways involved in anaerobic hydrocarbon oxidation and their ecological significance in contaminated environments. The findings provide theoretical insights and scientific evidence for bioremediation strategies in hydrocarbon-impacted ecosystems.

## 2. Materials and Methods

### 2.1. Site Description

**Figure 1:**
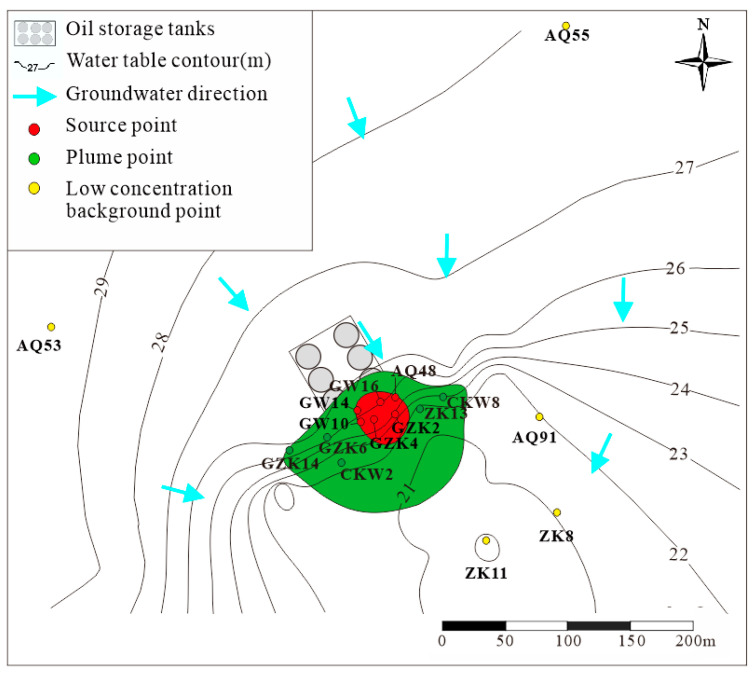
The sketch map of groundwater flow and sampling locations. The blue arrow donates the groundwater flow direction. The dots represent the sampling locations.

The study site is located near a gasoline storage tank area of a refinery in southern China, in operation since 1976. Groundwater contamination was first identified in 2019, revealing severe pollution by petroleum hydrocarbons (PHC) (Figure 1), primarily C_6_–C_9_ compounds (Supplementary Table S2) [18]. No remediation efforts had been undertaken as of the sample collection date. The groundwater table is approximately 4 m below the surface. The aquifer at the site primarily consists of red silty clay with a thickness of about 4 m. Beneath the aquifer lies a clay layer that acts as an aquiclude, with the total iron content in the aquifer approximately 4–5% [19]. Groundwater flows from northwest to southeast. Monitoring wells were installed along the groundwater flow direction, upstream, and around the storage tank area to monitor contaminants in the groundwater (Figure 1).

### 2.2. Sampling and Tests

(1) Groundwater collection

Samples were collected from groundwater wells in May 2022 using a sampler, following methods previously described [20,21]. For metagenomic analysis, 2 L of groundwater were collected into sterilized plastic containers. Groundwater for inorganic chemical analysis was collected into two 500 mL plastic containers, while groundwater for organic chemical analysis was collected into four 40 mL amber glass bottles with polytetrafluoroethylene (PTFE) seals. All samples were immediately refrigerated and transported to the laboratory as soon as possible.

DNA was extracted from 0.22 μm PTFE filters, which were used to filter 2 L of groundwater using an air pump under sterile conditions. The process was completed within 12 h, and the filters were stored at −80 °C until DNA extraction.

(2) Hydrochemical Measurements

In the laboratory, C_6_–C_9_ were measured using a gas chromatograph (GC) (SHIMADZU GC-2030, Shimadzu Corporation, Kyoto, Japan). Standard methods [22,23,24] were employed to measure hydrochemical parameters, including pH, chemical oxygen demand (CODcr), dissolved oxygen (DO), Fe^2+^, Mn^2+^, NO_3_^−^, SO_4_^2−^, and HCO_3_^−^.

(3) DNA Extraction, Sequencing, and Bioinformatics Analysis

DNA extraction was performed using the E.Z.N.A.^®^ DNA Kit (Omega Bio-tek, Norcross, GA, USA) following the manufacturer’s instructions. The metagenomic sequencing strategy involved the following steps:

The extracted DNA was purified and fragmented using the DNA shearing instrument (Covaris M220, Covaris Inc., Woburn, MA, USA) to an average fragment size of ~400 bp for paired-end library construction. Sequencing was performed on the Illumina HiSeq 4000 platform (Illumina Inc., San Diego, CA, USA) by Majorbio Bio-Pharm Technology Co., Ltd. (Shanghai, China), following previously established methods [21].

Illumina paired-end reads were processed using SeqPrep (v1.3, Open-source, GitHub, Abington, MA, USA) and Sickle (v1.33, Open-source, GitHub, USA) to remove adapter sequences and low-quality reads from the 3′ and 5′ ends. Metagenomic data were assembled using MEGAHIT (v 1.1.2, HKU-BGI Bioinformatics Algorithms Research Laboratory, Hong Kong, China), retaining contigs of ≥300 bp. Open reading frames (ORFs) were predicted using MetaGene (v2.3, Kyoto University, Kyoto, Japan) and translated into amino acid sequences. Predicted genes were clustered based on 95% sequence identity and 90% coverage, with the longest sequence in each cluster selected as the representative sequence to construct a non-redundant gene catalog. Representative sequences were annotated against the NCBI NR database using BLASTP (v2.14.0, National Center for Biotechnology Information, Bethesda, MD, USA) with an e-value threshold of 1 × 10^−5^. Functional genes were annotated using the KEGG enzyme database [25], and their relative abundances were expressed as enzyme annotations normalized to reads per million (RPM) [26].

### 2.3. Analytical Method

(1) Grouping

Groundwater samples from different locations were grouped based on their C_6_–C_9_ concentrations and the upstream-downstream relationships: Source Zone Group: Defined as groundwater samples containing non-aqueous phase liquid (NAPL) and located adjacent to the oil tanks. Plume Group: Defined as samples with C_6_–C_9_ concentrations between 0.15 mg/L and 8 mg/L, located outside the source zone. Background Group: Defined as samples with C_6_–C_9_ concentrations below 0.15 mg/L. (Since contaminants were detected in all areas of the refinery, the concentration threshold of 0.15 mg/L was established as the background value based on the grid-based background value survey conducted in the refinery area). According to this criterion, the groups were divided as follows: Background Group: AQ53, AQ55, ZK8, ZK11, and AQ91; Source Zone Group: GW14, GW10, GZK4, GZK2, AQ48, and GW16; and Plume Group: GZK14, CKW2, GZK6, ZK13, and CKW8.

(2) Analysis of Iron-Related Genes and Influencing Factors

Based on the study by Garber (2020) [3] and KEGG metabolic pathways, a total of 88 genes encoding key enzymes involved in four categories of iron metabolism—iron acquisition, regulation, reduction/oxidation, and storage—were identified (as shown in Supplementary Table S3). To investigate the environmental factors influencing the expression and functional potential of iron-related gene processes, an iron gene set representing the four categories of iron cycling functions was constructed by grouping iron-related genes based on their functions, the distribution of gene abundances across different functional categories was plotted under the grouped pollution regions, and inter-group gene abundance differences were analyzed. Additionally, the gene abundance data of different categories of iron-related genes from all sampling sites were integrated with the concentrations of various environmental factors. Correlation analyses were conducted using IBM SPSS Statistics version 26.0, resulting in a heatmap illustrating the correlations between the abundances of different categories of iron-related genes and multiple environmental factors. Principal Coordinate Analysis (PCoA) was conducted to examine the clustering or dispersion of microbial communities based on sample distances calculated using the Bray–Curtis distance algorithm. The significance of differences in microbial community composition and functional potential between the control and treatment groups was statistically evaluated using ANOSIM analysis.

(3) Microorganisms harboring iron-reducing genes and their community composition

Based on the studies by Shaw et al. (1992) [27], Chen et al. (2004) [28], Takai et al. (2001) [29], Hudson et al. (1993) [30], Stillman et al. (2001) [31], and Bou-Abdallah et al. (2014) [32], along with KEGG metabolic pathways, genes encoding iron-reducing enzymes were identified. Six specialized enzymes directly related to iron reduction processes were selected for analysis: EC 1.18.1.3 [27], EC 1.16.3.1 [28,29], EC 1.16.3.2 [30,31,32], EC 1.18.1.2 [33], EC 1.16.1.2 [34], and EC 1.16.1.7 [35]. In soil environments, these iron-reducing enzymes exhibit substrate specificity, particularly in the iron reduction processes. For example, EC 1.18.1.3 (ferredoxin-NAD+ reductase) specifically participates in the reduction of iron-sulfur proteins in anaerobic metabolism, while EC 1.16.3.1 (iron oxidase) plays crucial roles in the oxidation of Fe(II) and iron biomineralization. EC 1.16.3.2 (bacterial non-heme ferritin) is mainly involved in iron storage and may contribute to iron mineralization processes. EC 1.18.1.2 (ferredoxin-NADP+ reductase) is mainly associated with electron transfer in photosynthesis, relevant to specific photosynthetic microorganisms or plants. EC 1.16.1.2 (diferric-transferrin reductase) and EC 1.16.1.7 (ferric-chelate reductase) are involved in iron acquisition and transport, especially in the reduction of iron chelates. The specificity of these enzymes allows them to play distinct roles in various soil ecosystems, shaping iron biogeochemistry and microbial functional dynamics in soil ecosystems.

To further investigate the community characteristics of IRMs, the identified functional genes associated with iron-reducing reactions were aligned with the NR database for taxonomic analysis. Bar plots were constructed to illustrate the species-level abundance and community composition of IRMs across different sample groups (background, source zone, and contaminant plume). (1) A percentage distribution plot was generated to summarize the overall representation of iron-reducing microorganisms. Species-level community composition across different groups was further analyzed based on taxonomic classification. (2) Additionally, a functional gene abundance plot associated with iron-reducing reactions for IRMs was generated to supplement the analysis and visualize the abundance of genes associated with IRMs. Furthermore, previously reported iron-reducing bacterial species from the literature were integrated and compared with the site’s microbial database to identify known iron-reducing species. Additionally, the correlations between environmental factors (e.g., DO, NO_3_^−^, Fe^2+^, Mn^2+^, and SO_4_^2−^, and hydrocarbons) and both known and newly identified microorganisms associated with iron-reducing processes were investigated, revealing their ecological roles and adaptive strategies under varying environmental conditions in the contaminated aquifer.

### 2.4. Sequence Accession Numbers

The metagenomic data have been archived in the NCBI Sequence Read Archive (SRA) under BioProject accession number PRJNA1146180, encompassing samples SAMN43087885–SAMN43087928.

## 3. Results and Discussions

### 3.1. Hydrogeochemical Characterization of Groundwater

The concentrations of pollutants, electron acceptors, metabolic byproducts, and other hydrochemical parameters measured in May 2022 are summarized in Table 1. All contamination indicators confirm that the aquifer remains impacted by petroleum hydrocarbons, with no uncontaminated samples detected. Monitoring wells in the source zone include GW14, GW10, GZK4, GZK2, AQ48, and GW16; monitoring wells in the contaminant plume zone include GZK14, CKW2, GZK6, ZK13, CKW8, and CKW9; while background monitoring wells include AQ53, AQ55, ZK8, ZK11, and AQ91.

**Figure 2:**
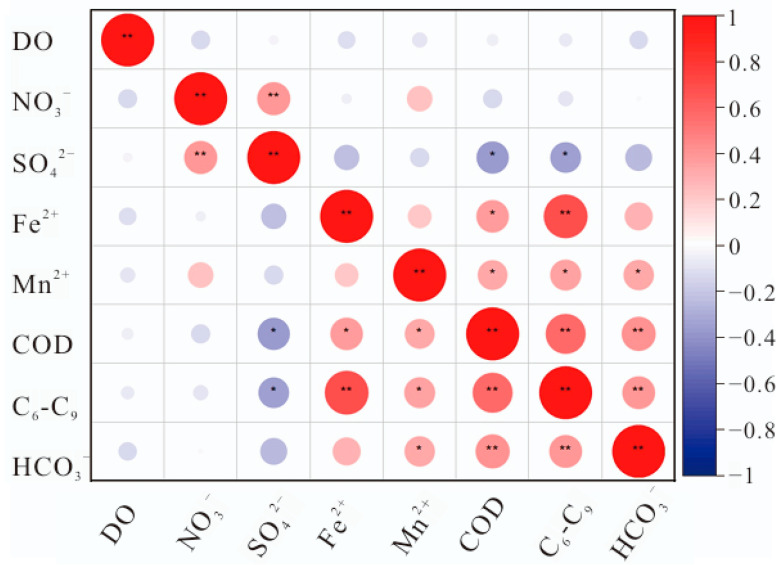
Correlation analysis between major pollutants and environmental factors. The asterisk (*) indicates a significant correlation (*p* < 0.05), while the double asterisk (**) indicates a highly significant correlation (*p* < 0.01).

Correlation analysis of the main pollutants (C_6_–C_9_ hydrocarbons) and primary electron acceptors at the site revealed that C_6_–C_9_ hydrocarbons were significantly positively correlated with iron and manganese concentrations, significantly negatively correlated with sulfate concentrations, and significantly positively correlated with HCO_3_^-^ concentrations. The correlation between COD and major electron acceptors was consistent with the results for C_6_–C_9_ hydrocarbons, as shown in Figure 2. This suggests that the degradation of pollutants is closely linked to the formation of iron and manganese, further supporting the conclusion that iron and manganese reduction likely dominates microbial degradation. It is hypothesized that the primary degradation process at the site involves iron-reducing hydrocarbon degradation, accompanied by sulfate and manganese reduction, which produces carbon dioxide as a metabolic byproduct.

In the aquifer, the biological degradation of pollutants is typically accompanied by the sequential consumption of a series of electron acceptors. Initially, oxygen and nitrate are preferentially consumed as electron acceptors, with their concentrations decreasing as the reaction progresses. Subsequently, the process enters the iron and manganese reduction phase, where microbial reduction of iron and manganese minerals occurs primarily through the direct contact mechanism, electron shuttle mechanism, and chelator mechanism [1]. During this phase, the concentrations of iron and manganese begin to rise, while the pollutants are mineralized into inorganic carbon. The absence of oxygen and nitrate at the site creates favorable conditions for iron and manganese-reducing hydrocarbon degradation.

Group statistics for the monitoring wells in the pollution source zone show that concentrations of C_6_–C_9_ hydrocarbons and chemical oxygen demand (COD) are significantly higher than those in the background and contaminant plume zones. The concentrations of electron acceptors Fe^2+^ and Mn^2+^ are significantly higher than those in the contaminant plume zone (see Supplementary Table S4 for additional data). The monitoring wells in the source zone exhibit higher concentrations of C_6_–C_9_ hydrocarbons and COD, indicating more severe petroleum hydrocarbon contamination compared to other wells. However, the contaminant plume zone also shows considerable pollution [36].

The higher concentrations of Fe^2+^ and Mn^2+^ in the source zone, compared to the background and plume zone wells, suggest that a dissimilatory metal reduction process may be occurring in the aquifer [4,37]. While most Fe^2+^ generated by the reduction of iron-bearing clay minerals and iron hydroxides is adsorbed onto aquifer materials or forms iron minerals that precipitate again, only about 2% of the generated Fe^2+^ may enter the solution [38,39]. Despite this, the concentration of Fe^2+^ remains significant.

This indicates that organic pollutants in the source zone are undergoing degradation and being mineralized into inorganic carbon, as evidenced by the significantly higher concentration of HCO_3_^−^ in the source zone compared to the background area (see Supplementary Figure S1). Combined with the degradation capacity calculations for the wells in the source zone (see Supplementary Table S5 for additional data. The derivation process is detailed in Supplementary Material: derivation process); the results suggest that iron-reduction-driven processes dominate the degradation. The distribution of iron closely resembles the distribution of petroleum hydrocarbon sources and plume zones (see Supplementary Figure S2 for additional details on Fe^2+^ (ferrous ion) contamination source zones and plumes).

### 3.2. Distribution Characteristics of Iron Genes and Iron Gene Neighborhoods

**Figure 3:**
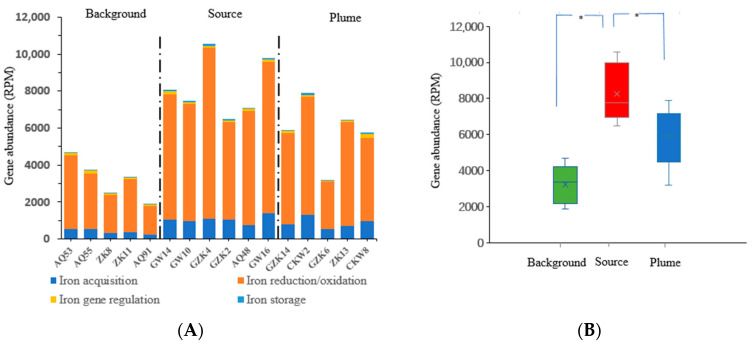
Abundance of various iron-related genes (**A**), boxplot of total iron gene abundance in different groups (**B**). The relative abundance of genes is expressed as reads per million (RPM) of annotated enzyme reads. The asterisk (*) indicates a significant difference between the two groups (*p* < 0.05).

Figure 3 provides a clear overview of the distribution and functional differences of iron-related genes across different regions. The abundance of these genes reflects the microbial activity in the iron biogeochemical cycle, particularly highlighting the differences between the source, plume, and background areas.

From Figure 3A, it is evident that the total iron-related gene abundance in the source area, especially the abundance of iron oxidation/reduction genes (orange bars), is higher than in both the background and plume areas. This suggests that microbial activity in the iron biogeochemical cycle is enhanced within the source zone. This high abundance may be associated with iron reduction environments and the iron-mediated redox processes associated with the biodegradation of organic pollutants, such as petroleum hydrocarbons. In contrast, the gene abundance in the background area is lower, particularly for iron oxidation/reduction genes, indicating weaker microbial activity, possibly due to lower contamination levels or a lack of driving forces for the iron cycle. The plume area exhibits an intermediate level of gene abundance, suggesting that pollutant dispersion may have activated certain functional genes in related microorganisms.

The boxplot in Figure 3B further supports the observed trends in total gene abundance, with the source area exhibiting the highest abundance of iron-related genes, the background area the lowest, and the plume area in between. This result aligns with the higher levels of pollution and the rich organic matter and carbon sources present in the source area, which promote microbial growth and the expression of functional genes. The relative abundance of specific iron-related genes also follows this pattern, as detailed in Supplementary Figure S3.

**Figure 4:**
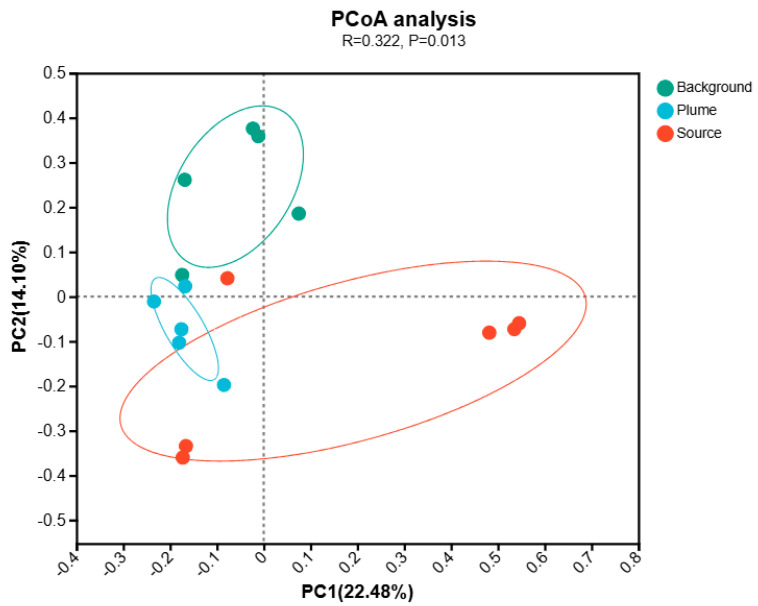
Principal Coordinate Analysis (PCoA) of Microbial Community Composition Across Sampling Areas. The circles represent confidence ellipses for different sample groups in the PCoA plot.

Additionally, the PCoA plot in Figure 4 clearly illustrates the compositional differentiation of microbial communities across different groups. Sample points from the source area cluster closely together, indicating a distinct microbial composition that significantly differs from those in both the background and plume areas. This suggests that the contamination conditions in the source area may have driven the selection of a distinct microbial community. The spatial separation of sample points further supports significant community differentiation among the source, background, and plume areas, with samples within each group forming tight clusters, while the groups themselves remain well separated. This pattern validates the classification scheme used in this study.

**Figure 5:**
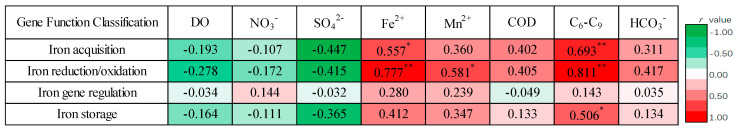
Correlation analysis between Gene abundance and environmental factors. The asterisk (*) indicates a significant correlation (*p* < 0.05), while the double asterisk (**) indicates a highly significant correlation (*p* < 0.01).

The analysis in Figure 5 shows a high positive correlation between various iron-related genes and environmental factors, such as Fe^2+^, Mn^2+^, C_6_–C_9_, COD, HCO_3_^−^, and CO_2_, while showing a negative correlation with DO, NO_3_^−^, and SO_4_^2−^. The PCoA/CCA analysis of microbial communities annotated with redox-specific iron genes further supports these findings (Supplementary Figure S4). Notably, genes related to iron oxidation/reduction and iron acquisition/transfer show a significant positive correlation with the concentrations of iron and C_6_–C_9_ hydrocarbons. Additionally, genes related to iron storage are significantly positively correlated with C_6_–C_9_ concentrations (Figure 5). These findings indicate that microbial iron reduction plays a crucial role in pollutant degradation. Specific iron-related genes, such as genes encoding the following enzymes EC 4.1.1.20, EC 4.2.1.118, EC 5.4.4.2, EC 7.2.2.20, EC 7.2.2.16, EC 7.6.2.8, EC 1.16.3.2, EC 2.7.13.3, EC 7.1.1.2, EC 1.14.15.20, and EC 1.14.15.25, show a significant positive correlation with iron and C_6_–C_9_ concentrations (See Supplementary Figure S5 for additional details).

Genes encoding the following enzymes EC 4.1.1.20, EC 4.2.1.118, EC 5.4.4.2, EC 7.2.2.20, EC 7.2.2.16, and EC 7.6.2.8 are primarily involved in microbial iron acquisition and transport, primarily involved in the uptake and transfer of iron within microorganisms. The gene encoding EC 1.16.3.2 functions in microbial iron storage. The distinctive triple iron-binding site of EcFtnA suggests its role in iron storage and oxidase activity [31]. Genes encoding the following enzymes EC 2.7.13.3, EC 7.1.1.2, EC 1.14.15.20, and EC 1.14.15.25 are associated with iron oxidation/reduction enzymes. For example, EC 2.7.13.3 is a histidine kinase that can sense the presence of iron ions and participate in the regulation of iron uptake and utilization. It may also be involved in the signal transduction pathways regulating iron redox processes. In *Geobacter*, iron reduction serves as a key metabolic pathway for energy generation, and histidine kinase may regulate the expression of associated metabolic genes [40]. EC 7.1.1.2, known as NADH: quinone reductase (H+-translocating), is a key component of the electron transport chain [41]. The gene encoding EC 1.14.15.20 encodes heme oxygenase (biliverdin formation, iron-dependent), which releases iron during the heme oxygenase reaction [42]. The gene encoding EC 1.14.15.25 encodes a p-umbelliferone methylmonooxygenase, an oxidoreductase involved in the degradation of aromatic compounds in certain *Pseudomonas* species [43]. All these genes are closely related to iron metabolism.

### 3.3. Microbial Community Composition and Its Relationship with Environmental Variables

**Figure 6:**
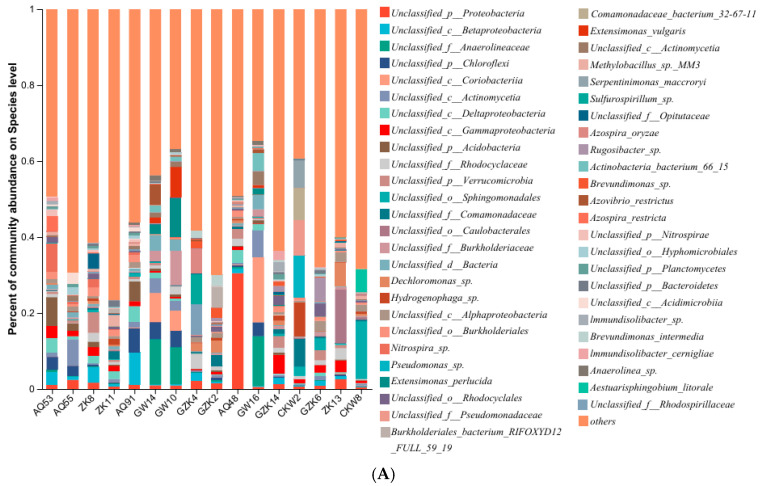

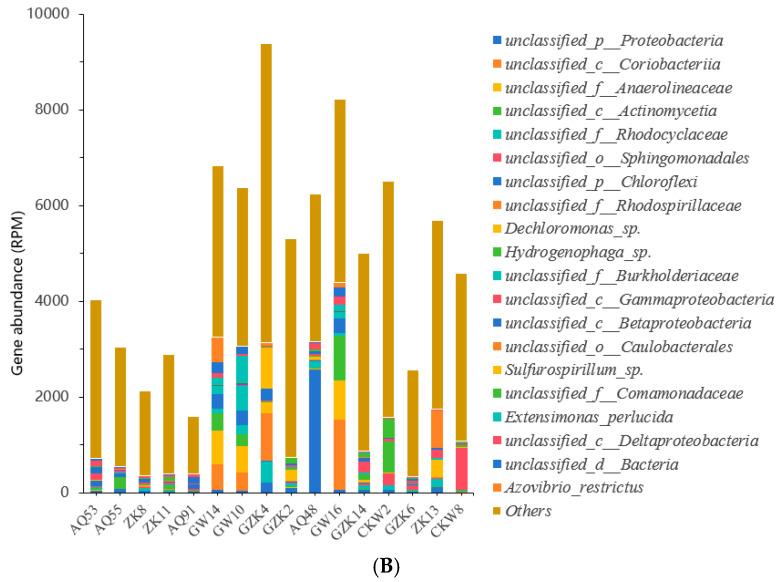
The microbial community abundance chart of the iron-reducing gene population (**A**) and the relative proportion chart of microorganisms in the iron-reducing gene population compared to the total microbial community (**B**) illustrate the relative proportions of different microbial species associated with the iron-reducing gene population in the samples. These proportions are calculated as the ratio of the incomplete reads annotated to microorganisms in the iron-reducing gene population to the total microbial reads in the sample, multiplied by 1,000,000. The unit is reads per million (RPM). Since the annotated microbial reads represent only a fragmentary collection of sequences for these microorganisms, the resulting ratio is smaller than the proportion of iron-reducing gene population genes to the total microbial population genes.

According to Figure 6A, which shows the taxonomic composition of microorganisms harboring iron-reducing related genes, the most abundant taxa are unclassified species within the Proteobacteria, widely distributed and particularly abundant in specific locations, such as AQ48. The second most abundant taxon is Betaproteobacteria, unclassified species within the Betaproteobacteria class of Proteobacteria, widely distributed, with relatively high concentrations in background samples (e.g., AQ53, ZK8, and AQ91). The third most abundant taxon is Anaerolineaceae, unclassified species within the Anaerolineaceae family of the Anaerolineales order, Anaerolineae class, and Chloroflexi phylum, predominantly found in source samples such as GW14, GW10, and GW16. Additionally, unclassified species within the Chloroflexi also exhibit high abundance, primarily in both background (e.g., AQ53, AQ55, and AQ91) and source locations (e.g., GW14, GW10, and GW16). Lastly, unclassified species within the Coriobacteriia class of the Actinobacteria are notably abundant in source locations GW14, GW10, and GW16.

According to Figure 6B, the top five microbial community abundances for iron-reducing related genes range from 1435.0 RPM to 3459.1 RPM. A total of 7290 microbial species associated with redox-active iron genes were identified, compared to 49,272 total species. Despite comprising only 14.80% of the total species, these species exhibit high relative abundance, indicating their ecological significance. The key iron-reducing bacteria at the site include unclassified species within the Proteobacteria phylum (including species within the Rhodocyclaceae family), unclassified species within the Coriobacteriia class of the Actinobacteria phylum, and unclassified species within the Actinomycetales order. Additionally, bacteria from the Chloroflexi phylum, such as species within the Anaerolineaceae family, are also major components of the microbial community (see Supplementary Figure S6 for details). Among identifiable species, *Azovibrio restrictus* contributes 0.91% to the iron-reducing gene population and 0.76% to the total microbial community. Since these proportions are close, it can be inferred that iron-reducing bacteria dominate the microbial community at this site.

Unclassified bacterial species within the phylum Proteobacteria, including those from the class *Betaproteobacteria* [44] and its families *Burkholderiaceae* [45] and *Rhodocyclaceae* [46], play a crucial role in the degradation of polycyclic aromatic hydrocarbons (PAHs) and other aromatic compounds. These species are widely involved in petroleum hydrocarbon remediation and are closely linked to iron redox cycling. Studies have shown that microorganisms from *Betaproteobacteria* were highly abundant in iron mats exposed to hydrocarbons and significantly contributed to the degradation of low-molecular-weight PAHs [44]. Similarly, members of the order Coriobacteriia [47] within the phylum Actinobacteria and the order Actinomycetales within the same phylum have been associated with the breakdown of complex organic compounds. Relevant literature suggests that Actinomycetales can utilize iron as an electron acceptor [48,49]. Although the iron-reducing capabilities of Coriobacteriia remain unconfirmed, their metabolic potential for complex organic compound degradation implies a possible indirect correlation with iron reduction.

Furthermore, bacteria from the phylum Chloroflexi, particularly those from the family Anaerolineaceae [50], specialize in degrading complex organic compounds under anaerobic conditions. They indirectly participate in petroleum hydrocarbon remediation and iron cycling, demonstrating multifunctionality in the restoration of polluted environments.

Specific microbial species, such as *Sulfurospirillum* sp. have been identified as highly efficient metal-reducing microorganisms, with multiple studies demonstrating their role in Fe(III) reduction and anaerobic hydrocarbon biodegradation through electron transfer mechanisms [1,51,52]. Additionally, *Extensimonas perlucida* has been detected in petroleum-contaminated environments, where its high abundance is significantly correlated with Fe^2+^ and organic compound concentrations. Metagenomic data indicate that this species harbors genes related to hydrocarbon degradation, suggesting its potential involvement in the anaerobic breakdown of petroleum hydrocarbons [53].

**Figure 7:**
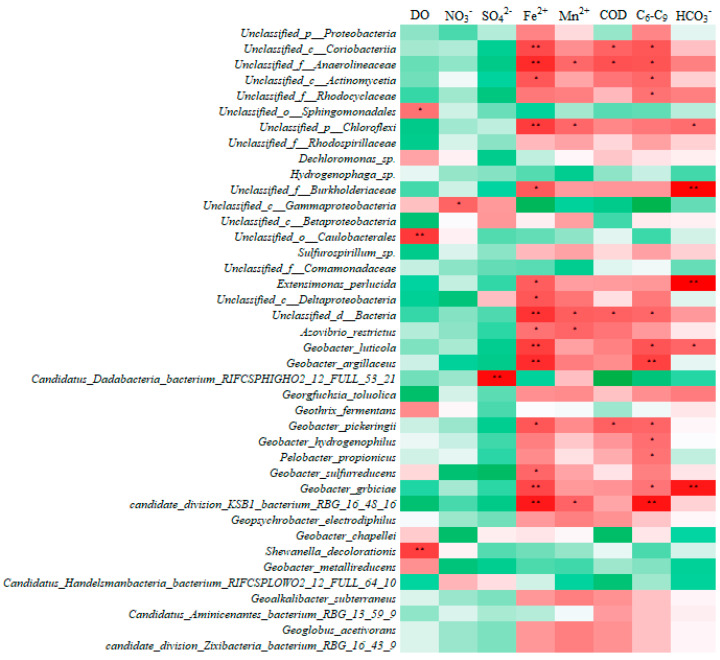
Correlation Heatmap Between Microbial Species and Environmental Factors. The asterisk (*) indicates a significant correlation (*p* < 0.05), while the double asterisk (**) indicates a highly significant correlation (*p* < 0.01).

Unclassified bacterial species in the phylum Proteobacteria, as well as species from the orders Actinomycetales and Coriobacteriia within the phylum Actinobacteria, and bacteria from the phylum Chloroflexi (such as the family Anaerolineaceae), show significant positive correlations with environmental factors, such as Fe^2+^, Mn^2+^, COD, and C_6_–C_9_ hydrocarbons, as shown in Figure 7. These correlations suggest a potential role in iron reduction and hydrocarbon degradation, warranting further functional validation [16,54]. Previous studies have shown that organic compounds can enhance the metabolic activity of iron-reducing bacteria [55], potentially explaining the observed positive correlation between iron concentration and microbial abundance in this study. During petroleum hydrocarbon bioremediation, iron redox processes may accelerate microbial degradation pathways [5], further underscoring the importance of iron availability in shaping microbial community dynamics.

Some bacterial species, such as *unclassified_o_Sphingomonadales* and *Hydrogenophaga* sp., show minimal correlation with Fe^2+^ and C_6_–C_9_ hydrocarbons. Although these species can utilize petroleum hydrocarbons to carry out hydrocarbon degradation facilitated by iron reduction, they preferentially thrive in iron-limited and toxic environments. As a result, they may not fully exert their metabolic functions in anaerobic environments with high iron concentrations. Therefore, the impact of iron concentration on their growth and metabolism may be limited. Furthermore, microorganisms typically exist in communities, and the degradation of petroleum hydrocarbons and iron metabolism may be carried out by multiple species working together. The direct effects of certain species may mask the indirect effects of others, leading to insignificant correlations.

Several previously identified iron-reducing species were detected in the metagenomic dataset, including *Geobacter luticola*, *Geobacter sulfurreducens*, *Geothrix fermentans*, and *Geobacter argillaceus*. These species are known to play crucial roles in petroleum hydrocarbon degradation and metal cycling by using iron as an electron acceptor [6]. Across the iron-associated microbial community, a total of 20 bacterial species were identified, accounting for 0.38% of the total abundance, with read counts ranging from 0.04 RPM to 116.3 RPM (see Supplementary Figure S7 for more details). The abundance of these iron-reducing species showed a significant positive correlation with the concentrations of Fe and C_6_–C_9_ in the environment. This suggests that, while these currently identified species represent a small fraction of the total community, many more iron-reducing bacteria remain undiscovered.

## 4. Conclusions

In this study, metagenomic sequencing technology was applied to groundwater from an iron-rich aquifer, revealing the presence of more than 7000 species of iron-reducing microorganisms (IRMs), including several previously well-characterized iron-reducing species (e.g., *Geobacter luticola* and *Geobacter sulfurreducens*). However, the majority of these IRMs were not found in existing iron-reducing microbial databases. Some of them, such as *Sulfurospirillum* sp. and *Extensimonas perlucida*, could be taxonomically classified at the species level, while most were only annotated as unclassified bacteria. In the contamination source zone, petroleum hydrocarbon pollution and iron-rich aquifer conditions facilitated the proliferation of these microbial communities, driving anaerobic hydrocarbon degradation predominantly mediated by microbial iron reduction. This study enhances our understanding of iron reduction coupled hydrocarbon degradation mechanisms and provides theoretical support for the bioremediation of petroleum-contaminated sites.

## Figures and Tables

**Table 1:** Water Chemistry Parameters in Different Sampling Locations of the Contaminated Aquifer (Low-Concentration background, Source, and Plume Zones).

Location	Low-Concentration Background					Source						Plume				
Sample ID	AQ53	AQ55	ZK8	ZK11	AQ91	GW14	GW10	GZK4	GZK2	AQ48	GW16	GZK14	CKW2	GZK6	ZK13	CKW8
DO (mg/L)	0.70	0.62	2.13	1.59	1.08	1.13	0.45	0.17	0.76	0.79	1.10	0.98	1.16	1.27	3.85	3.24
NO_3_^−^ (mg/L)	0.89	3.99	0.89	0.89	2.66	1.33	1.77	1.77	1.77	0.89	1.77	5.76	0.89	3.10	2.21	1.77
SO_4_^2−^ (mg/L)	54.00	12.00	10.00	7.00	27.00	2.00	3.00	3.00	5.00	7.00	1.00	20.00	3.00	27.00	0.00	1.00
Fe^2+^ (mg/L)	0.42	0.22	0.35	0.22	0.24	11.10	11.69	8.73	1.81	12.05	12.29	0.82	0.62	0.03	0.13	0.22
Mn^2+^ (mg/L)	0.94	0.09	0.78	0.20	0.20	1.10	0.78	0.94	0.52	0.67	0.78	0.25	0.25	0.20	0.36	0.52
COD (mg/L)	4.10	8.30	23.00	35.00	1.00	39.00	29.00	26.90	37.30	12.80	39.00	14.20	12.00	11.30	19.00	15.00
C_6_–C_9_ (mg/L)	0.05	0.12	0.04	0.00	0.03	16.00	16.01	20.07	16.01	22.48	25.46	2.10	7.03	4.27	0.63	2.67
HCO_3_^−^ (mg/L)	250.60	137.83	363.37	400.96	375.90	563.85	2042.40	789.39	613.97	438.55	501.20	476.14	162.89	275.66	426.02	501.20

## Data Availability

The original contributions presented in this study are included in the article/Supplementary Materials. Further inquiries can be directed to the corresponding author.

## Supplementary Materials

The following supporting information can be downloaded at: https://www.mdpi.com/article/10.3390/microorganisms13020433/s1, Figure S1: Boxplot of bicarbonate (HCO_3_^−^) concentration in different groups; Figure S2: Fe^2+^ (Ferrous Ion) Contamination Source Zones and Plumes; Figure S3: Relative Abundance of Specific Functional Genes Related to Iron Acquisition, Iron Gene Regulation, Iron Reduction/Oxidation, and Iron Storage; Figure S4: CCA Analysis of Environmental Variables and Microbial Communities; Figure S5: Correlation Heatmap Between Genes Involved in Iron Metabolism and Hydrochemical Parameters; Figure S6: Species-Level Community Structure Diagram; Figure S7: Gene Abundance of Known Isolated and Cultivated Strains in Microbial Populations Annotated with Iron-reducing Genes; Table S1: List of IRMs; Table S2: Concentration of Organic Pollutant Components; Table S3: Functional Iron Genes and Corresponding Enzymes Involved in Iron Acquisition, Gene Regulation, Reduction/Oxidation, and Storage; Table S4: Significance Value (*p*) of Differences Between Source Zone and Contamination Plume; Table S5: Degradation Capacity of Electron Acceptors in Single Wells (mg/L).
