# Supplementary material for: Metagenomic Insights into the Abundance of Iron-Reducing Microorganisms in a Petroleum-Contaminated Iron-Rich Aquifer
Source: Microorganisms. 2025 Feb 17;13(2):433. doi: 10.3390/microorganisms13020433 (PMC11858104; doi:10.3390/microorganisms13020433)
Supplement: Supplementary file 1 [file microorganisms-13-00433-s001.zip › microorganisms-3434698-supplementary.pdf]

Supplementary Information
for
Metagenomic Insights into the Abundance of Iron-Reducing Microorganisms in a Petroleum-Contaminated Iron-Rich Aquifer

He Di [1,2,3,4], Min Zhang [1,3,*], Zhuo Ning [1,3], Changli Liu [1,3,4], Ze He [1,3], Shuaiwei Wang [1,3], Siyu Kong [1,3], Shuang Gan [1,3], Weichao Sun [1,3], Zhe Xu [1,3], Jinjin Ti [1,3]

[1] Institute of Hydrogeology and Environmental Geology, Chinese Academy of Geological Sciences, Shijiazhuang 050061, China; 3020190008@email.cugb.edu.cn (H.D.); ningzhuozhuo@163.com (Z.N.); liuchangli@vip.163.com (C.L.); heze25@163.com (Z.H.); tairan_w@163.com (S.W.); 13273188379@163.com (S.K.); ganshuang2016@163.com (S.G.); 17730568088@163.com (W.S.); xu991102021@163.com (Z.X.); tijinjin22@mails.ucas.ac.cn (J.T.)
[2] School of Chinese Academy of Geological Sciences, China University of Geosciences (Beijing), Beijing 100086, China
[3] Key Laboratory of Groundwater Remediation of Hebei Province & China Geological Survey, Zhengding 050083, China
[4] Key Laboratory of Water Cycle and Ecological Geological Processes, Xiamen 361021, China

*Corresponding author
E-mail: zhangmin@mail.cgs.gov.cn; Tel.: +86-0311-67598605

Supporting Information: 24 pages, 7 Figures, 5 Tables

1.Analytical methods

(1) Boxplot of bicarbonate (HCO_3^-) concentration in different groups. This figure presents a boxplot of HCO_3^- concentrations across different groups (source zone, background, and contaminant plume) to visualize the distribution and variation in bicarbonate levels within each group. See Figure S1: Boxplot of bicarbonate (HCO_3^-) concentration in different groups.

(2) The contamination map was constructed using kriging interpolation based on the distribution data of ferrous ions (Fe^{2+}) from sampling points, and the plume boundaries were delineated. See Figure S2: Fe^{2+} (Ferrous Ion) Contamination Source Zones and Plumes.

(3) The abundance of iron metabolism-related genes was annotated using metagenomic data and compared with KEGG. The relative abundance of functional genes involved in iron acquisition, regulation, reduction/oxidation, and storage was normalized by the total gene abundance. The results are presented as a heatmap, where each row of data is first centered (by subtracting the mean to make the dataset have a mean of zero) and then scaled (adjusted to a specific range, e.g., [-2, 2]). The colors in the heatmap represent the relative abundance of each gene per row, with the color scale indicated by the key at the bottom right of the figure. Gray boxes represent genes that were not detected. See Figure S3: Relative Abundance of Specific Functional Genes Related to Iron Acquisition, Iron Gene Regulation, Iron Reduction/Oxidation, and Iron Storage

(4) CCA analysis and plotting were performed using the R vegan package (version 2.4.3). Canonical Correspondence Analysis (CCA) was employed to explore the relationships between environmental factors (such as DO, NO_3^-, Fe^{2+}, Mn^{2+}, SO_4^{2-}, C_6-C_9 hydrocarbons, COD, HCO_3^-, CO_2) and microbial communities or functions, as well as the correlations among these factors. See Figure S4: CCA Analysis of Environmental Variables and Microbial Communities.

(5) Spearman's rank correlation analysis was used to assess the relationships between iron metabolism genes and environmental factors, including DO, NO_3^-, SO_4^{2-}, Fe^{2+}, Mn^{2+}, COD, C_6-C_9 hydrocarbons, and HCO_3^-. See Figure S5: Correlation Heatmap Between Genes Involved in Iron Metabolism and Hydrochemical Parameters

(6) Species-Level Community Structure. At the species level, bar plots were used to illustrate the abundance and community composition of microorganisms at each sampling site. A percentage distribution plot was also created. See Figure S6: Species-Level Community Structure Diagram.

(7) Gene Abundance in Cultivated Strains.This figure illustrates the abundance of known iron-reducing bacterial species. Previously reported iron-reducing species from the literature were integrated and compared with the microbial database from the study site. A bar plot was generated to display the relative abundance of these species across the samples. See Figure S7: Gene Abundance of Known Isolated and Cultivated Strains in Microbial Populations Annotated with Iron Redox Genes.

(8) A comprehensive list of IRMs was compiled based on their roles in iron reduction. Information was curated from publicly available databases and literature. See Table S1.

(9) Concentration of Organic Pollutant Components. Obtained by sampling and analyzing organic pollutants in water samples. See Table S2.

(10)Based on the study by Garber (2020) [3]and KEGG metabolic pathways, a total of 88 key enzymes involved in iron metabolism were identified. A detailed list of functional genes and their corresponding enzymes related to iron metabolism (iron acquisition, regulation, reduction/oxidation, and storage) was created by integrating functional annotations with enzyme databases (e.g., KEGG). See Table

S3: Functional Iron Genes and Corresponding Enzymes Involved in Iron Acquisition, Gene Regulation, Reduction/Oxidation, and Storage.

(11) Significance Value (P) of Differences Between Source Zone and Contamination Plume. This table presents the results of non-parametric tests (e.g., Mann-Whitney U test) used to evaluate statistical differences in environmental and microbial parameters between the source zone and contaminant plume. See Table S4.

(12) Degradation Capacity of Electron Acceptors. See Table S5.

The degradation capacity of oxygen (DO), nitrate (NO_3^-), iron [Fe(III)], manganese [Mn(IV)], and sulfate (SO_4^{2-}) was calculated using benzene as a representative pollutant. The utilization coefficients F for DO, NO_3^-, Mn, and SO_4^{2-} were 3.14, 4.9, 10.7 and 4.7, respectively. The formula for single-well degradation capacity is as follows:

$$Q = (C - C_0)/F \quad (1)$$

Where Q represents the single-well degradation capacity, C is the concentration of the electron acceptor in the well, and C_0 is the average concentration of the selected background points.

Based on this formula, the single-well degradation capacities for DO, NO_3^-, [Mn(IV)], and SO_4^{2-}were calculated. When iron [Fe(III)] is used as the electron acceptor, its utilization coefficient F is 21.8, and the ratio of liquid-phase iron produced to solid-phase iron is 0.0222 [1]. Therefore, the single-well degradation capacity for Fe(III) can be expressed as[2]:

$$Q_{Fe} = (C_{Fe} - C_{Fe0})/F \cdot 0.0222 \quad (2)$$

2. Figures

Figure S1. Boxplot of bicarbonate (HCO_3^-) concentration in different groups

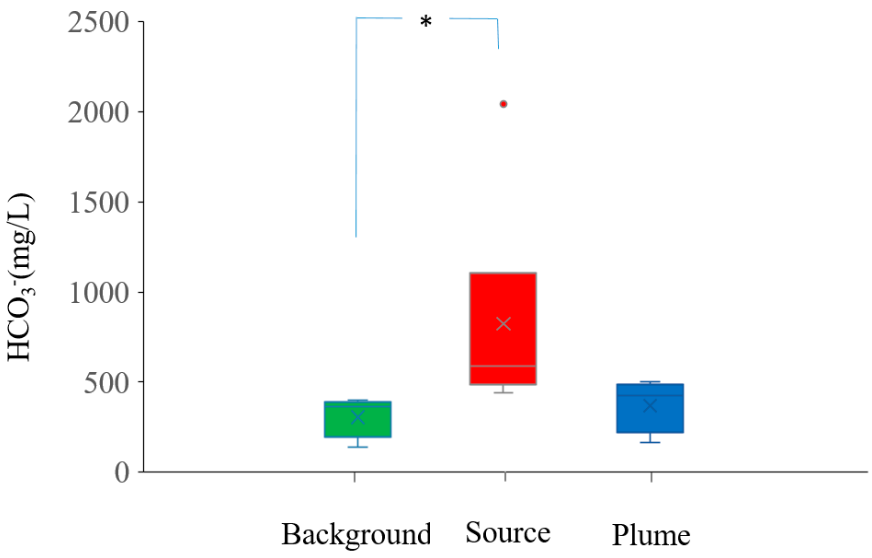

Figure S2. Fe^{2+} (Ferrous Ion) Contamination Source Zones and Plumes

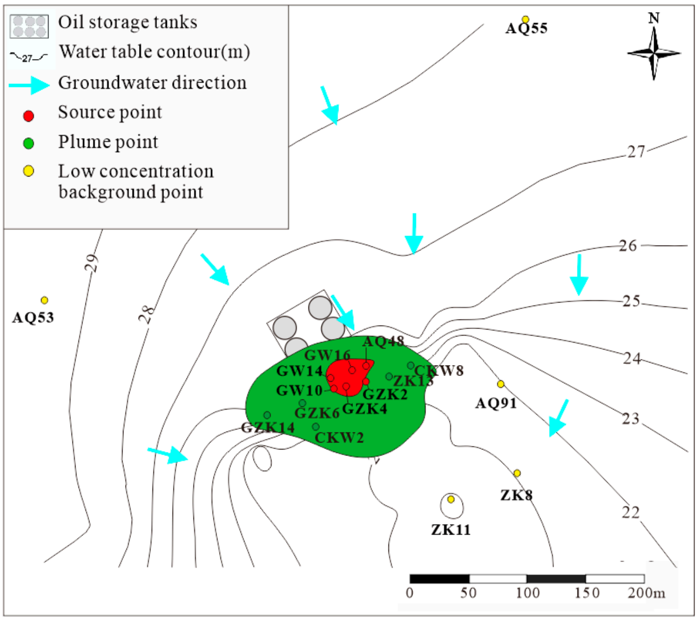

Figure S3. Relative Abundance of Specific Functional Genes Related to Iron Acquisition, Iron Gene Regulation, Iron Reduction/Oxidation, and Iron Storage.The heatmap presents the normalized relative abundance of these genes after centering and scaling by row. Centering involves subtracting the mean of the data to create a dataset with a mean of zero, while scaling adjusts the data to a specific range (in this case, [-2, 2]). The colors are scaled relative to each row, as indicated by the color key at the bottom right of the figure. Gray boxes represent genes that were not detected.

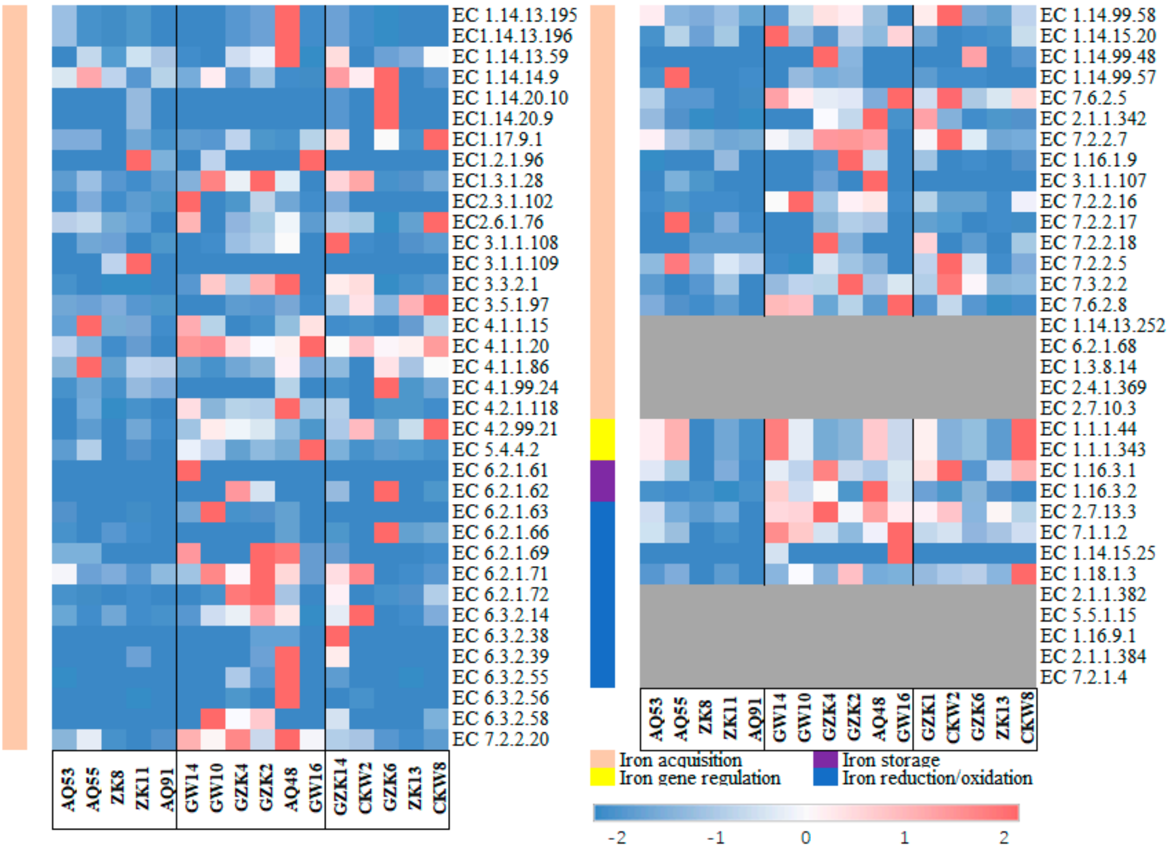

Figure S4. CCA Analysis of Environmental Variables and Microbial Communities.

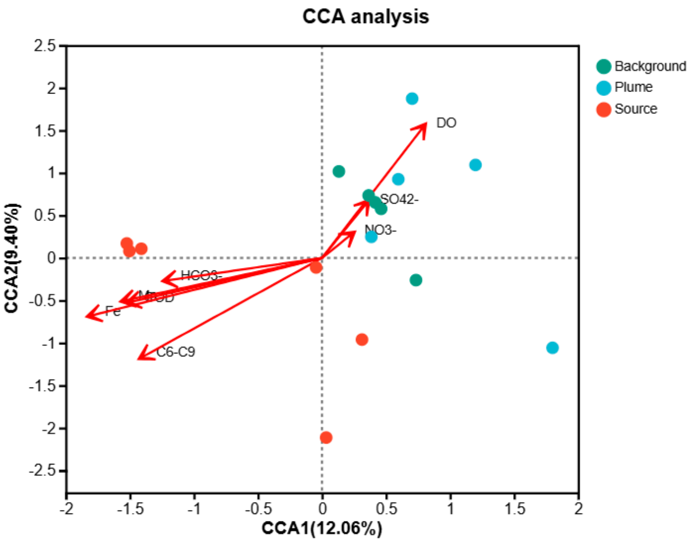

Figure S5. Correlation Heatmap Between Genes Involved in Iron Metabolism and Hydrochemical Parameters.The heatmap illustrates the correlations between genes involved in iron metabolism and hydrochemical parameters. Red indicates positive correlations, while blue indicates negative correlations. Asterisks (*) denote significance levels (*, $p < 0.05$; **, $p < 0.01$).

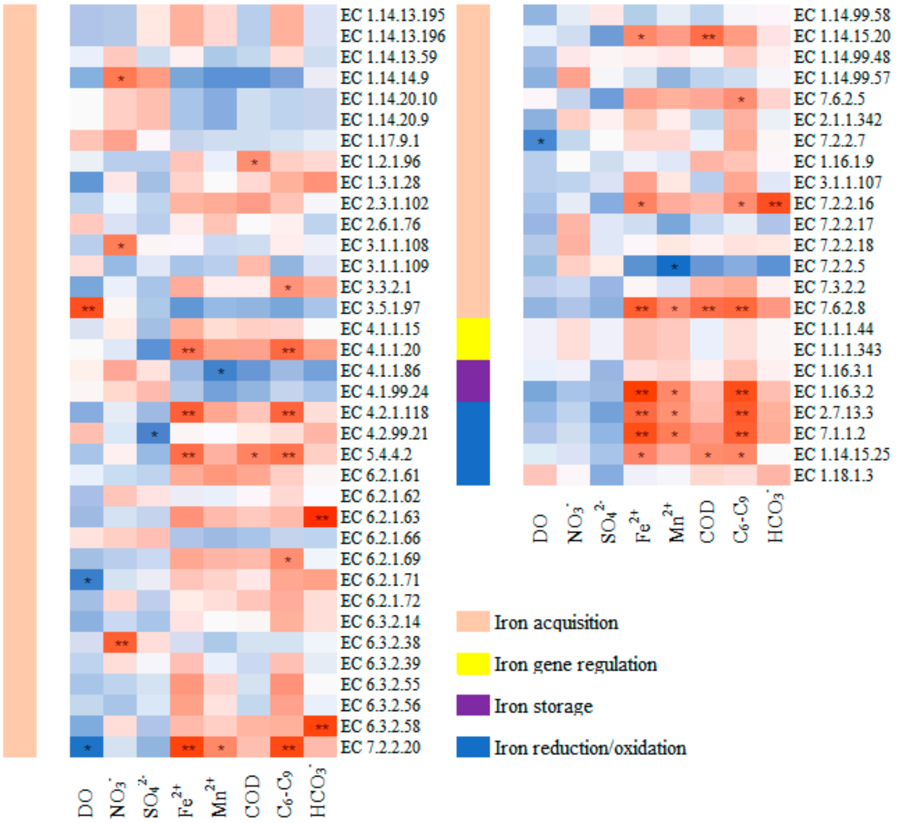

Figure S6. Species-Level Community Structure Diagram

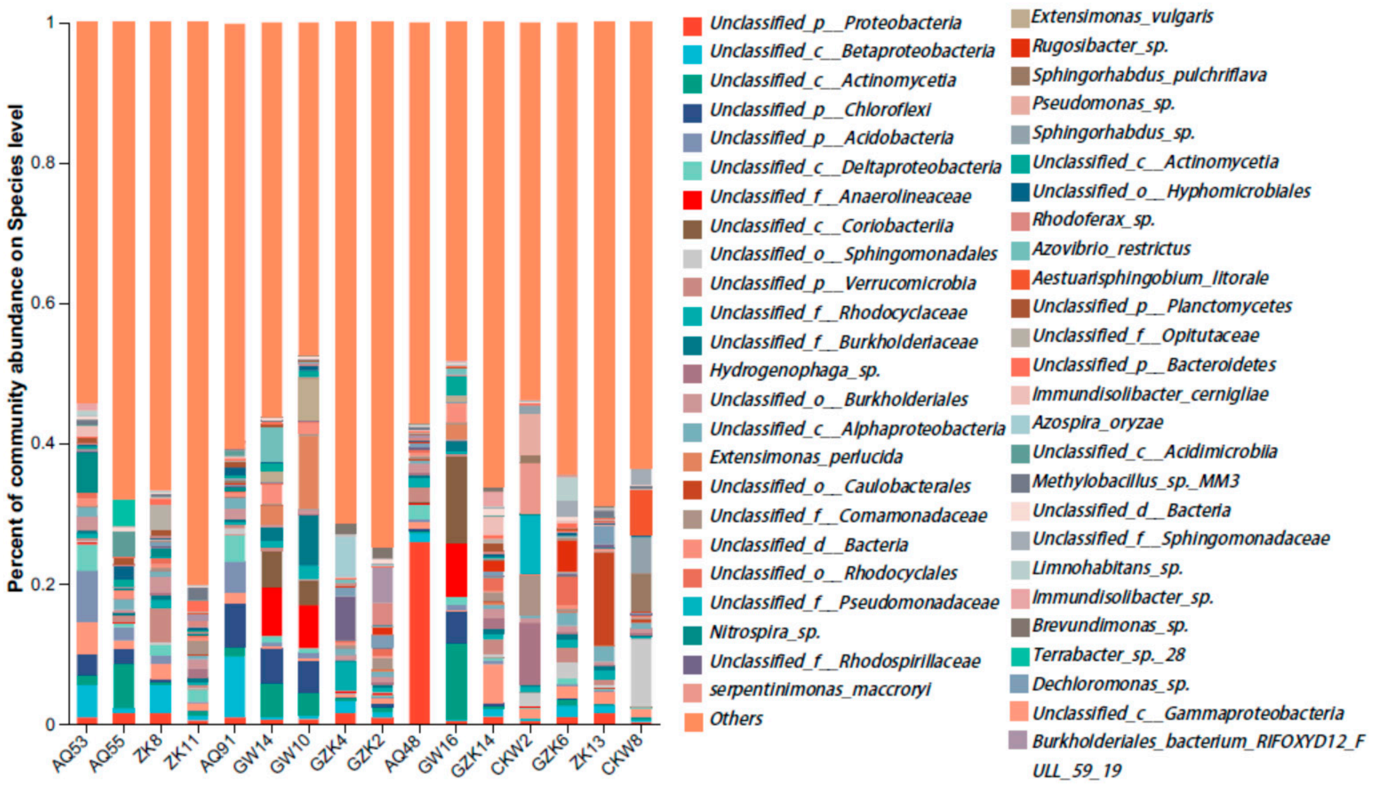

Figure S7. Gene Abundance of Known Isolated and Cultivated Strains in Microbial Populations Annotated with Iron-reducing Genes.Gene abundance is expressed in reads per million (RPM), representing the number of sequencing reads annotated as enzymes per million reads, which indicates the relative abundance of the genes.

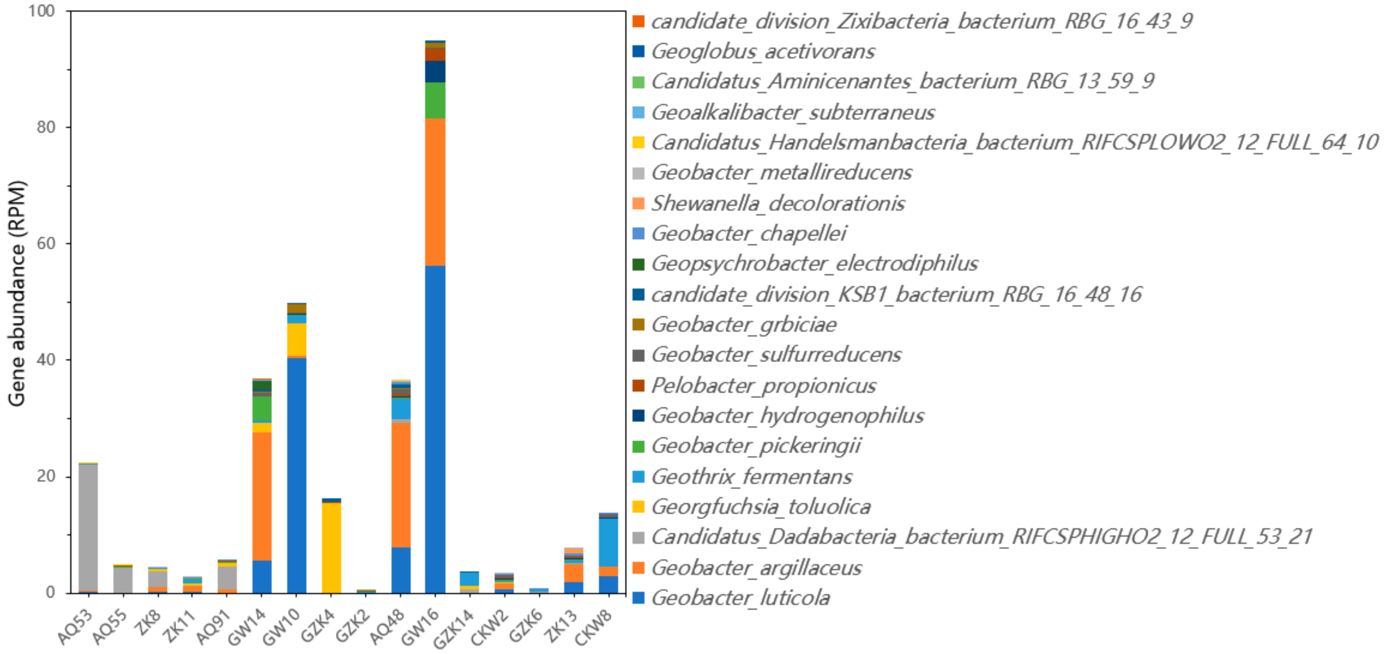

3.Tables

Table S1. List of IRMs

1	Aggregatibacter actinomycetemcomitans Y4 Scfld0	Garber et al., 2020 [3]
2	Bacillus anthracis SK-102	
3	Bacillus subtilis subsp. subtilis str. 168	
4	Capnocytophaga ochracea DSM 7271	
5	Chlorobium tepidum TLS	
6	Dechloromonas aromatica RCB	
7	Escherichia coli K-12	
8	Ferroplasma acidarmanus fer1	
9	Geobacter bemidjiensis	
10	Geobacter sulfurreducens PCA	
11	Listeria monocytogenes 10403S	
12	Magnetospirillum magneticum AMB-1	
13	Mariprofundus ferrooxydans PV-1	
14	Porphyromonas gingivalis W83	
15	Prochlorococcus marinus subsp. marinus str. CCMP1375	
16	Pseudomonas aeruginosa PAO1	
17	Rhodobacter ferrooxidans SW2 ctg00005	
18	Rhodoferax ferrireducens T118	
19	Rhodopseudomonas palustris TIE-1	
20	Shewanella oneidensis MR-1	
21	Streptococcus mutans UA159	
22	Sulfolobus metallicus DSM 6482	
23	Synechocystis sp. IPPAS B-1465	
24	Desulfovibrio ferrophilus	
25	Acaryochloris marina MBIC11017	
26	Acaryochloris marina CCMEE 5410	
27	Marinimicrobia bacterium JGI 0000039-D08	
28	Candidate division KSB1 bacterium RBG_16_48_16	
29	Candidate division Zixibacteria bacterium RBG_16_43_9	
30	Candidatus Aminicenantes bacterium RBG_13_59_9	
31	Candidatus Schekmanbacteria bacterium RBG_13_48_7	
32	Candidatus Portnoybacteria bacterium RBG_19FT_COMBO_36_7	

33	Candidatus Nealsonbacteria bacterium RIFCSPLOWO2_01_FULL_38_120	
34	Candidatus Kuenenbacteria bacterium CG1_02_38_13	
35	Candidatus Omnitrophica bacterium CG1_02_44_16	
36	Candidatus Nitrospira defluvii	
37	Candidatus Caldatribacterium californiense OP9-cSCG	
38	Candidatus Dadabacteria bacterium RIFCSPHIGHO2_12_FULL_53_21	
39	Candidatus Lindowbacteria bacterium RIFCSPLOWO2_12_FULL_62_27	
40	Candidatus Tectomicrobia bacterium RIFCSPLOWO2_12_FULL_69_37	
41	Candidatus Raymondbacteria bacterium RIFOXYB2_FULL_49_35	
42	Candidatus Rokubacteria bacterium 13_2_20CM_2_70_11	
43	Candidatus Handelsmanbacteria bacterium RIFCSPLOWO2_12_FULL_64_10	
44	Archaeon GW2011_AR4	
45	Borrelia burgdorferi	
46	Treponema pallidum	
47	Pelosinus fermentans	Esther et al., 2015 [4]
48	Geoglobus acetivorans sp.nov.	
49	Geothrix fermentans gen.nov.sp. Nov	
50	Geoalkalibactersubterraneussp. nov.	
51	Deferribacter thermophilus	
52	Bacillussubterraneus	
53	Acidiphilium cryptum JF-5*	
54	Bacillus pseudormus MCO2	
55	Shewanella amazonensis sp.nov.	
56	Shewanella putrefaciens IR-1	
57	Shewanella sp.HN-41	
58	Shewanella putrefaciens CN-32	
60	Shewanella baltica W3-6-1	
61	Shewanella sp.PV-4	
62	Shewanella peizotolerans WP3	
63	Shewanella decolorationis	
64	Shewanella frigidimarinai	
65	Shewanella gelidimarina	
66	Shewanella loihica	

67	Shewanella pealeana	
68	Geobacter bemidjiensis sp.nov.	
69	Geobacter psychrophilus sp. nov.	
70	Geobacter bremensis sp.nov.	
71	Geobacter pelophilus sp.nov.	
72	Geobacter daltonii	
73	Geobacter lovleyi sp. nov.strain SZ	
74	Geobacter metallireducens	
75	Geobacter luticola	
76	Geobacter pickeringii	
77	Geobacter argillaceus	
78	Geobacter sulfurreducens	
79	Geobacter uraniireducens	
80	Desulfitobacterium aromaticivorans UKTLT	Castro et al., 2022 [5]; Rooney-Varga et al., 1999[6]; Farkas et al., 2016[7].
81	Ferroglobus placidus	
82	Geobacter chapellei	
83	Geobacter grbiciae	
84	Geobacter grbiciae TACP-21	
85	Geobacter grbiciae TACP-5	
86	Geobacter hydrogenophilus	
87	Geobacter strain Ben	
88	Geobacter hiogenes	
89	Geobacter toluenoxydans TMJ1	
90	Geopsychrobacter electrodiphilus	
91	Georgfuchsia toluolica	
92	Hydrogenophaga sp. PYR1	
93	Pelobacter acidigallici	
94	Pelobacter propionicus	
95	Trichococcus alkaliphilus	

Table S2. Concentration of Organic Pollutant Components

SampleID	GW10	GW14	GW16	AQ48
Benzene (mg/L)	1.07	3.53	4.52	-
Toluene (mg/L)	0.06	0.18	0.22	-
Ethylbenzene (mg/L)	-	0.29	0.29	-
m-/p-Xylene (mg/L)	-	0.23	0.34	-
o-Xylene (mg/L)	-	-	-	-
1,2,3-Trichloropropane(mg/L)	-	-	0.51	-
Methyl tert-butyl ether (mg/L)	1.25	4.02	5.14	
C_6-C_9 hydrocarbons (mg/L)	3.07	6.39	9.50	0.61
C_{10}-C_{40} hydrocarbons (mg/L)	0.02	0.03	0.05	0.08

Table S3. Functional Iron Genes and Corresponding Enzymes Involved in Iron Acquisition, Gene Regu-lation, Reduction/Oxidation, and Storage

Function	EC Number	EC Name	Reference
Iron acquisition	1.14.99.58	heme oxygenase (biliverdin-IX-beta and delta-forming); pigA (gene name)	Ratliff et al., 2011 [8]
	1.3.8.14	L-prolyl-[peptidyl-carrier protein] dehydrogenase; pigA (gene name); bmp3 (gene name); pltE (gene name); redW (gene name); (L-prolyl)-[peptidyl-carrier protein]:electron-transfer flavoprotein oxidoreductase	Thomas et al., 2002 [9]
	1.14.15.20	heme oxygenase (biliverdin-producing, ferredoxin); HO1 (gene name); HY1 (gene name); HO3 (gene name); HO4 (gene name); pbsA1 (gene name)	Montgomery et al., 2002; Sugishima et al., 2004 [10, 11]
	1.14.99.48	heme oxygenase (staphylobilin-producing); haem oxygenase (ambiguous); heme oxygenase (decyclizing) (ambiguous); heme oxidase (ambiguous); haem oxidase (ambiguous); heme oxygenase (ambiguous); isdG (gene name); isdI (gene name)	Reniere et al., 2010 [12]
	1.14.99.57	heme oxygenase (mycobilin-producing); mhuD (gene name)	Chim et al., 2010 [13]
	7.6.2.5	ABC-type heme transporter; heme-transporting ATPase	Ramseier et al., 1991; Jekabsons et al., 1995; Saier et al., 1998 [14-16]
	2.1.1.342	anaerobilin synthase; chuW (gene name)	LaMattina et al., 2016 [17]

Function	EC Number	EC Name	Reference
	7.2.2.7	ABC-type Fe3+ transporter; Fe3+-transporting ATPase	Angerer et al., 1992; Kuan et al., 1995 [18, 19]
	6.2.1.62	3,4-dihydroxybenzoate---[aryl-carrier protein] ligase; asbC (gene name)	Pfleger et al., 2007 [20]
	4.2.1.118	3-dehydroshikimate dehydratase	Fox et al., 2008; Pfleger et al., 2008 [21, 22]
	6.2.1.66	glyine---[glycyl-carrier protein] ligase; dhbF (gene name); sfmB (gene name)	Li et al., 2008 [23]
	1.3.1.28	2,3-dihydro-2,3-dihydroxybenzoate dehydrogenase; 2,3-DHB dehydrogenase; 2,3-dihydro-2,3-dihydroxybenzoate:NAD+ oxidoreductase	Young et al., 1969 [24]
	5.4.4.2	isochorismate synthase; MenF	van Tegelen et al., 1999 [25] Young et al., 1969 [24] Dahm et al., 1998 [26] Daruwala et al., 1996 [27]
	6.2.1.71	2,3-dihydroxybenzoate---[aryl-carrier protein] ligase; entE (gene name); vibE (gene name); dhbE (gene name); angE (gene name)	Khalil et al., 2010 [28]
	6.3.2.39	aerobactin synthase; iucC (gene name)	Oves-Costales et al., 2009 [29]
	1.14.13.59	L-lysine N6-monooxygenase (NADPH); lysine N6-hydroxylase; L-lysine 6-monooxygenase (NADPH) (ambiguous)	MACHEROUX et al., 1993 [30]

Function	EC Number	EC Name	Reference
	1.2.1.96	4-hydroxybenzaldehyde dehydrogenase (NADP+); p-hydroxybenzaldehyde dehydrogenase (ambiguous); pchA (gene name)	Chen et al., 2014 [31]
	4.2.99.21	isochorismate lyase; salicylate biosynthesis protein pchB; pyochelin biosynthetic protein PchB; isochorismate pyruvate lyase	Kerbarh et al., 2005 [32]
	6.2.1.68	L-glutamate---[L-glutamyl-carrier protein] ligase; ambE (gene name)	Rojas Murcia et al., 2015 [33]
	6.2.1.61	salicylate---[aryl-carrier protein] ligase; pmsE (gene name); pchD (gene name)	Sattely et al., 2008 [34]
	6.2.1.69	L-cysteine---[L-cysteinyl-carrier protein] ligase; pchE (gene name); pchF (gene name); angR (gene name)	Quadri et al., 1999 [35]
	1.17.9.1	4-methylphenol dehydrogenase (hydroxylating); pchCF (gene names); p-cresol-(acceptor) oxidoreductase (hydroxylating); p-cresol methylhydroxylase; 4-cresol dehydrogenase (hydroxylating)	Johannes et al., 2008 [36]
	1.14.13.195	L-ornithine N5-monooxygenase (NADPH); CchB; ornithine hydroxylase; EtcB; PvdA; Af-OMO; dffA (gene name)	Robbel et al., 2011 [37]
	1.14.13.196	L-ornithine N5-monooxygenase [NAD(P)H]; SidA (ambiguous)	Franceschini et al., 2012 [38]
	7.2.2.20	ABC-type Zn2+ transporter; Zn2+-transporting ATPase; Zn2+ ABC transporter; znuABC (gene names)	Hantke et al., 2005 [39] Patzer et al., 1998[40]
	2.6.1.76	diaminobutyrate---2-oxoglutarate transaminase; L-2,4-diaminobutyrate:2-ketoglutarate 4-aminotransferase; 2,4-diaminobutyrate 4-aminotransferase; diaminobutyrate aminotransferase; DABA aminotransferase; DAB aminotransferase; EctB; diaminibutyric	Kuhlmann Anne et al., 2002 [41]

Function	EC Number	EC Name	Reference
		acid	
	1.14.14.9	4-hydroxyphenylacetate 3-monooxygenase; p-hydroxyphenylacetate 3-hydroxylase; 4-hydroxyphenylacetic acid-3-hydroxylase; p-hydroxyphenylacetate hydroxylase (FAD); 4 HPA 3-hydroxylase; p-hydroxyphenylacetate 3-hydroxylase (FAD); HpaB	Louie et al., 2003 [42]
	4.1.99.24	L-tyrosine isonitrile synthase; pvcA (gene name)	Chang et al., 2017 [43]
	1.14.20.9	L-tyrosine isonitrile desaturase; pvcB (gene name)	Zhu et al., 2015 [44]
	1.14.20.10	L-tyrosine isonitrile desaturase/decarboxylase; pvcB (gene name)	Zhu et al., 2015 [44]
	3.5.1.97	acyl-homoserine-lactone acylase; acyl-homoserine lactone acylase; AHL-acylase; AiiD; N-acyl-homoserine lactone acylase; PA2385 protein; quorum-quenching AHL acylase; quorum-quenching enzyme; QuiP	Sio Charles et al., 2006 [45]
	4.1.1.20	diaminopimelate decarboxylase; diaminopimelic acid decarboxylase; meso-diaminopimelate decarboxylase; DAP-decarboxylase; meso-2,6-diaminoheptanedioate carboxy-lyase	Denman et al., 1955 [46]
	4.1.1.15	glutamate decarboxylase; L-glutamic acid decarboxylase; L-glutamic decarboxylase; cysteic acid decarboxylase; L-glutamate alpha-decarboxylase; aspartate 1-decarboxylase; aspartic alpha-decarboxylase; L-aspartate-alpha-decarboxylase; gamma-glutamate	Nakano et al., 1971 [47]
	4.1.1.86	diaminobutyrate decarboxylase; DABA DC; L-2,4-diaminobutyrate decarboxylase; L-2,4-diaminobutanoate carboxy-lyase	Ikai et al., 1997 [48]
	6.3.2.38	N2-citryl-N6-acetyl-N6-hydroxylysine synthase; Nalpha-citryl-Nepsilon-acetyl-Nepsilon-hydroxylysine synthase; iucA (gene name)	Oves-Costales et al., 2009 [29]
	6.3.2.55	2-[(L-alanin-3-ylcarbamoyl)methyl]-3-(2-aminoethylcarbamoyl)-2-hydroxypropanoate	Cheung et al.,

Function	EC Number	EC Name	Reference
		synthase; sbnF (gene name)	2009 [49]
	6.3.2.56	staphyloferrin B synthase; sbnC (gene name)	Cheung et al., 2009 [49]
	1.14.13.252	putrescine N-hydroxylase; alcA (gene name); pubA (gene name); fbsI (gene name)	Lyons et al., 2022 [50]
	6.3.2.58	D-ornithine---citrate ligase; sfnaD (gene name)	Cotton et al., 2009 [51]
	3.3.2.1	isochorismatase; 2,3-dihydro-2,3-dihydroxybenzoate synthase; 2,3-dihydroxy-2,3-dihydrobenzoate synthase; 2,3-dihydroxy-2,3-dihydrobenzoic synthase	Young et al., 1969 [24]
	6.3.2.14	enterobactin synthase; N-(2,3-dihydroxybenzoyl)-serine synthetase; 2,3-dihydroxybenzoylserine synthetase; 2,3-dihydroxybenzoate---serine ligase	Shaw-Reid et al., 1999 [52]
	3.1.1.107	apo-salmochelin esterase; iroE (gene name)	Lin et al., 2005 [53]
	3.1.1.108	iron(III)-enterobactin esterase; fes (gene name); pfeE (gene name); enterochelin hydrolase; enterochelin esterase; ferric enterobactin esterase	Perraud et al., 2018 [54]
	7.3.2.2	ABC-type phosphonate transporter; phosphonate-transporting ATPase (ambiguous)	Saier et al., 1998 [16]
	2.7.10.3	bacterial tyrosine kinase; BY-kinase; bacterial protein tyrosine kinase	Chao et al., 2014 [55]
	7.2.2.16	ABC-type ferric hydroxamate transporter; iron(III) hydroxamate transporting ATPase; iron(III) hydroxamate ABC transporter; fhuCDB (gene names)	Speziali Craig et al., 2006 [56]
	7.2.2.5	ABC-type Mn2+ transporter; ABC-type manganese permease complex; manganese-transporting ATPase (ambiguous); ABC-type manganese transporter	Kolenbrander Paul et al., 1998

Function	EC Number	EC Name	Reference
			[57]
	7.6.2.8	ABC-type vitamin B12 transporter; BtuCDF; vitamin B12 ABC transporter; vitamin B12-transporting ATPase	Saier et al., 1998 [16] Kuan et al., 1995 [19] Friedrich et al., 1986 [58]
	2.4.1.369	enterobactin C-glucosyltransferase; iroB (gene name)	Fischbach et al., 2005 [59]
	7.2.2.18	ABC-type ferric citrate transporter; ferric citrate transporting ATPase; ferric citrate ABC transporter; fecBCDE (gene names)	Banerjee et al., 2016 [60]
	7.2.2.17	ABC-type ferric enterobactin transporter; ferric enterobactin transporting ATPase; ferric enterobactin ABC transporter; fepBCDG (gene names)	Shea et al., 1991 [61]
	1.16.1.9	ferric-chelate reductase (NADPH); ferric chelate reductase (ambiguous); iron chelate reductase (ambiguous); NADPH:Fe3+-EDTA reductase; NADPH-dependent ferric reductase; yqjH (gene name); Fe(II):NADP+ oxidoreductase	Miethke et al., 2011 [62]
Iron gene regulation	1.1.1.44	phosphogluconate dehydrogenase (NADP+-dependent, decarboxylating); phosphogluconic acid dehydrogenase; 6-phosphogluconic dehydrogenase; 6-phosphogluconic carboxylase; 6-phosphogluconate dehydrogenase (decarboxylating); 6-phospho-D-gluconate dehydrog	Zamboni et al., 2004 [63]
	1.1.1.343	phosphogluconate dehydrogenase (NAD+-dependent, decarboxylating); 6-PGDH (ambiguous); gntZ (gene name); GNDl	Zamboni et al., 2004 [63]
Iron reduction/oxidation	5.5.1.15	terpentedienyl-diphosphate synthase; terpentedienol diphosphate synthase; Cyc1; clerodadienyl diphosphate synthase; terpentedienyl-diphosphate lyase (decyclizing)	Eguchi et al., 2003 [64]
	1.16.9.1	iron:rusticyanin reductase; Cyc2 (ambiguous)	Quatrini et al.,

Function	EC Number	EC Name	Reference
			2009 [65]
	2.1.1.384	[methyl-Co(III) methoxylated-aromatic-compound-specific corrinoid protein]---tetrahydromethanopterin methyltransferase; mtoA (gene name)	Welte et al., 2021 [66]
	7.2.1.4	tetrahydromethanopterin S-methyltransferase; tetrahydromethanopterin methyltransferase; mtrA-H (gene names); cmtA (gene name); N5-methyltetrahydromethanopterin---coenzyme M methyltransferase; 5-methyl-5,6,7,8-tetrahydromethanopterin:2-mercaptoethane	Vepachedu Venkata et al., 2012 [67]
	7.1.1.2	NADH:ubiquinone reductase (H+-translocating); ubiquinone reductase (ambiguous); type 1 dehydrogenase; complex 1 dehydrogenase; coenzyme Q reductase (ambiguous); complex I (electron transport chain); complex I (mitochondrial electron transport); comp	Wikström et al., 2012 [68]
	1.14.15.25	p-cymene methyl-monooxygenase; cymAa (gene name); cymA (gene name); p-cymene methyl hydroxylase	Dutta et al., 2010 [69]
	1.18.1.3	ferredoxin---NAD+ reductase; ferredoxin-nicotinamide adenine dinucleotide reductase; ferredoxin reductase (ambiguous); NAD+-ferredoxin reductase; NADH-ferredoxin oxidoreductase; reductase, reduced nicotinamide adenine dinucleotide-ferredoxin; ferred	SHAW et al., 1992 [70]
	2.7.13.3	histidine kinase; EnvZ; histidine kinase (ambiguous); histidine protein kinase (ambiguous); protein histidine kinase (ambiguous); protein kinase (histidine) (ambiguous); HK1; HP165; Sln1p	Yoshimi et al., 2004; Kowluru et al., 2002 [71, 72]
	2.1.1.382	methoxylated aromatic compound---corrinoid protein Co-methyltransferase; mtoB (gene name); mtvB (gene name); vdmB (gene name)	Welte et al., 2021; Kurth et al., 2021; Pierce et al., 2008 [66, 73, 74]
Iron storage	1.16.3.1	ferroxidase; ceruloplasmin; caeruloplasmin; ferroxidase I; iron oxidase; iron(II):oxygen oxidoreductase; ferro:O2 oxidoreductase; iron II:oxygen oxidoreductase; hephaestin; HEPH	Chen et al., 2004; Takai et al., 2001

Function	EC Number	EC Name	Reference
			[75, 76]
	1.16.3.2	bacterial non-heme ferritin; FtnA; HuHF	Hudson et al., 1993; Stillman et al., 2001; Bou-Abdallah et al., 2014 [77-79]

Table S4. Significance Value (P) of Differences Between Source Zone and Contamination Plume

DO	NO_3^-	SO_4^{2-}	Fe^{2+}	Mn^{2+}	COD	C_6-C_9
0.02	0.158	0.406	0.000	0.009	0.021	0.000

Table S5. Degradation Capacity of Electron Acceptors in Single Wells (mg/L)

Sites	GW14	GW10	GZK4	GZK2	AQ48	GW16
DO degradation capacity	0	0.07	0.16	0	0	0
NO_3^- degradation capacity	0.23	0.14	0.14	0.14	0.32	0.14
SO_4^{2-} degradation capacity	6.60	6.38	6.38	5.96	5.53	6.81
Mn(IV) degradation capacity	0.05	0.02	0.04	0.00	0.01	0.02
Fe(III) degradation capacity	24.72	26.08	19.27	3.40	26.89	27.44
Proportion of Iron Degradation Capacity	0.78	0.80	0.74	0.36	0.82	0.80

References

1. Chapelle, F.H.;P.M. Bradley;M.A. Thomas, and P.B. McMahon, Distinguishing Iron-Reducing from Sulfate-Reducing Conditions. *Groundwater*, **2009**, *47*, 300-305.
2. Ning, Z.;M. Zhang;Z. He, et al., Spatial Pattern of Bacterial Community Diversity Formed in Different Groundwater Field Corresponding to Electron Donors and Acceptors Distributions at a Petroleum-Contaminated Site. *Water*, **2018**, *10*, 842.
3. Garber, A.I.;K.H. Nealson;A. Okamoto, et al., FeGenie: a comprehensive tool for the identification of iron genes and iron gene neighborhoods in genome and metagenome assemblies. *Frontiers in Microbiology*, **2020**, *11*, 499513.
4. Esther, J.;L.B. Sukla;N. Pradhan, and S. Panda, Fe (III) reduction strategies of dissimilatory iron reducing bacteria. *Korean J. Chem. Eng.*, **2015**, *32*, 1-14.
5. Castro, A.R.;G. Martins;A.F. Salvador, and A.J. Cavaleiro, Iron Compounds in Anaerobic Degradation of Petroleum Hydrocarbons: A Review. *Microorganisms*, **2022**, *10*, 2142.
6. Rooney-Varga, J.N.;R.T. Anderson;J.L. Fraga, et al., Microbial Communities Associated with Anaerobic Benzene Degradation in a Petroleum-Contaminated Aquifer. *Appl. Environ. Microbiol.*, **1999**, *65*, 3056-3063.
7. Farkas, M.;S. Szoboszlay;T. Benedek, et al., Enrichment of dissimilatory Fe(III)-reducing bacteria from groundwater of the Siklós BTEX-contaminated site (Hungary). *Folia Microbiologica*, **2017**, *62*, 63-71.
8. Ratliff, M.;W. Zhu;R. Deshmukh, et al., Homologues of Neisserial Heme Oxygenase in Gram-Negative Bacteria: Degradation of Heme by the Product of the<i>pigA</i> Gene of <i>Pseudomonas aeruginosa</i>. *J. Bacteriol.*, **2001**, *183*, 6394-6403.
9. Thomas, M.G.;M.D. Burkart, and C.T. Walsh, Conversion of L-Proline to Pyrrolyl-2-Carboxyl-S-PCP during Undecylprodigiosin and Pyoluteorin Biosynthesis. *Chem. Biol.*, **2002**, *9*, 171-184.
10. Montgomery, B.L. and J.C. Lagarias, Phytochrome ancestry: sensors of bilins and light. *Trends Plant Sci.*, **2002**, *7*, 357-366.
11. Sugishima, M.;C.T. Migita;X. Zhang, et al., Crystal structure of heme oxygenase-1 from cyanobacterium Synechocystis sp. PCC 6803 in complex with heme. *Eur. J. Biochem.*, **2004**, *271*, 4517-4525.
12. Reniere, M.L.;G.N. Ukpabi;S.R. Harry, et al., The IsdG-family of haem oxygenases degrades haem to a novel chromophore. *Mol. Microbiol.*, **2010**, *75*, 1529-1538.
13. Chim, N.;A. Iniguez;T.Q. Nguyen, and C.W. Goulding, Unusual Diheme Conformation of the Heme-Degrading Protein from Mycobacterium tuberculosis. *J. Mol. Biol.*, **2010**, *395*, 595-608.

14. Ramseier, T.M.;H.V. Winteler, and H. Hennecke, Discovery and sequence analysis of bacterial genes involved in the biogenesis of c-type cytochromes. *J. Biol. Chem.*, **1991**, *266*, 7793-7803.
15. Jekabsons, W. and W. Schuster, orf250 encodes a second subunit of an ABC-type heme transporter in Oenothera mitochondria. *Molecular and General Genetics MGG*, **1995**, *246*, 166-173.
16. Saier, M.H., *Molecular Phylogeny as a Basis for the Classification of Transport Proteins from Bacteria, Archaea and Eukarya*, in *Adv. Microb. Physiol.*, Poole, R.K., Editor; Academic Press, 1998; 40. p. 81-136.
17. LaMattina, J.W.;D.B. Nix, and W.N. Lanzilotta, Radical new paradigm for heme degradation in <i>Escherichia coli</i> O157:H7. *Proceedings of the National Academy of Sciences*, **2016**, *113*, 12138-12143.
18. Angerer, A.;B. Klupp, and V. Braun, Iron transport systems of Serratia marcescens. *J. Bacteriol.*, **1992**, *174*, 1378-1387.
19. Kuan, G.;E. Dassa;W. Saurin, et al., Phylogenetic analyses of the ATP-binding constituents of bacterial extracytoplasmic receptor-dependent ABC-type nutrient uptake permeases. *Res. Microbiol.*, **1995**, *146*, 271-278.
20. Pfleger, B.F.;J.Y. Lee;R.V. Somu, et al., Characterization and Analysis of Early Enzymes for Petrobactin Biosynthesis in Bacillus anthracis. *Biochemistry*, **2007**, *46*, 4147-4157.
21. Fox, D.T.;K. Hotta;C.-Y. Kim, and A.T. Koppisch, The Missing Link in Petrobactin Biosynthesis: asbF Encodes a (−)-3-Dehydroshikimate Dehydratase. *Biochemistry*, **2008**, *47*, 12251-12253.
22. Pfleger, B.F.;Y. Kim;T.D. Nusca, et al., Structural and functional analysis of AsbF: Origin of the stealth 3,4-dihydroxybenzoic acid subunit for petrobactin biosynthesis. *Proceedings of the National Academy of Sciences*, **2008**, *105*, 17133-17138.
23. Li, L.;W. Deng;J. Song, et al., Characterization of the Saframycin A Gene Cluster from Streptomyces lavendulae NRRL 11002 Revealing a Nonribosomal Peptide Synthetase System for Assembling the Unusual Tetrapeptidyl Skeleton in an Iterative Manner. *J. Bacteriol.*, **2008**, *190*, 251-263.
24. Young, I.G. and F. Gibson, Regulation of the enzymes involved in the biosynthesis of 2,3-dihydroxybenzoic acid in Aerobacter aerogenes and Escherichia coli. *Biochimica et Biophysica Acta (BBA) - General Subjects*, **1969**, *177*, 401-411.
25. van Tegelen, L.o.J.P.;P.R.H. Moreno;A.F. Croes, et al., Purification and cDNA Cloning of Isochorismate Synthase from Elicited Cell Cultures of Catharanthus roseus. *Plant Physiol.*, **1999**, *119*, 705-712.
26. Dahm, C.;R. Müller;G. Schulte, et al., The role of isochorismate hydroxymutase genes entC and menF in enterobactin and menaquinone biosynthesis in Escherichia coli. *Biochimica et Biophysica Acta (BBA) - General Subjects*, **1998**, *1425*, 377-386.
27. Daruwala, R.;O. Kwon;R. Meganathan, and M.E.S. Hudspeth, A new isochorismate synthase specifically involved in menaquinone (vitamin K2) biosynthesis encoded by the menF gene. *FEMS Microbiol. Lett.*, **1996**, *140*, 159-163.
28. Khalil, S. and P.D. Pawelek, Enzymatic Adenylation of 2,3-Dihydroxybenzoate Is Enhanced by a Protein−Protein Interaction

between Escherichia coli 2,3-Dihydro-2,3-dihydroxybenzoate Dehydrogenase (EntA) and 2,3-Dihydroxybenzoate-AMP Ligase (EntE). *Biochemistry*, **2011**, *50*, 533-545.

29. Oves-Costales, D.;N. Kadi, and G.L. Challis, The long-overlooked enzymology of a nonribosomal peptide synthetase-independent pathway for virulence-conferring siderophore biosynthesis. *Chem. Commun. (Cambridge, U. K.)*, **2009**, 6530-6541.

30. MACHEROUX, P.;H.J. PLATTNER;A. ROMAGUERA, and H. DIEKMANN, FAD and substrate analogs as probes for lysine N6-hydroxylase from Escherichia coli EN 222. *Eur. J. Biochem.*, **1993**, *213*, 995-1002.

31. Chen, Y.-F.;H. Chao, and N.-Y. Zhou, The catabolism of 2,4-xylenol and p-cresol share the enzymes for the oxidation of para-methyl group in Pseudomonas putida NCIMB 9866. *Appl. Microbiol. Biotechnol.*, **2014**, *98*, 1349-1356.

32. Kerbarh, O.;A. Ciulli;I. Howard Nigel, and C. Abell, Salicylate Biosynthesis: Overexpression, Purification, and Characterization of Irp9, a Bifunctional Salicylate Synthase from Yersinia enterocolitica. *J. Bacteriol.*, **2005**, *187*, 5061-5066.

33. Rojas Murcia, N.;X. Lee;P. Waridel, et al., The Pseudomonas aeruginosa antimetabolite L -2-amino-4-methoxy-trans-3-butenoic acid (AMB) is made from glutamate and two alanine residues via a thiotemplate-linked tripeptide precursor. *Frontiers in Microbiology*, **2015**, *6*.

34. Sattely, E.S. and C.T. Walsh, A Latent Oxazoline Electrophile for N−O−C Bond Formation in Pseudomonine Biosynthesis. *J. Am. Chem. Soc.*, **2008**, *130*, 12282-12284.

35. Quadri, L.E.N.;T.A. Keating;H.M. Patel, and C.T. Walsh, Assembly of the Pseudomonas aeruginosa Nonribosomal Peptide Siderophore Pyochelin: In Vitro Reconstitution of Aryl-4,2-bisthiazoline Synthetase Activity from PchD, PchE, and PchF. *Biochemistry*, **1999**, *38*, 14941-14954.

36. Johannes, J.;A. Bluschke;N. Jehmlich, et al., Purification and Characterization of Active-Site Components of the Putative p-Cresol Methylhydroxylase Membrane Complex from Geobacter metallireducens. *J. Bacteriol.*, **2008**, *190*, 6493-6500.

37. Robbel, L.;V. Helmetag;T.A. Knappe, and M.A. Marahiel, Consecutive Enzymatic Modification of Ornithine Generates the Hydroxamate Moieties of the Siderophore Erythrochelin. *Biochemistry*, **2011**, *50*, 6073-6080.

38. Franceschini, S.;M. Fedkenheuer;N.J. Vogelaar, et al., Structural Insight into the Mechanism of Oxygen Activation and Substrate Selectivity of Flavin-Dependent N-Hydroxylating Monooxygenases. *Biochemistry*, **2012**, *51*, 7043-7045.

39. Hantke, K., Bacterial zinc uptake and regulators. *Curr. Opin. Microbiol.*, **2005**, *8*, 196-202.

40. Patzer, S.I. and K. Hantke, The ZnuABC high-affinity zinc uptake system and its regulator Zur in Escherichia coli. *Mol. Microbiol.*, **1998**, *28*, 1199-1210.

41. Kuhlmann Anne, U. and E. Bremer, Osmotically Regulated Synthesis of the Compatible Solute Ectoine in Bacillus pasteurii and Related Bacillus spp. *Appl. Environ. Microbiol.*, **2002**, *68*, 772-783.

42. Louie, T.M.;X.S. Xie, and L. Xun, Coordinated Production and Utilization of FADH2 by NAD(P)H−Flavin Oxidoreductase and

4-Hydroxyphenylacetate 3-Monooxygenase. *Biochemistry*, **2003**, *42*, 7509-7517.

43. Chang, W.-c.;D. Sanyal;J.-L. Huang, et al., In Vitro Stepwise Reconstitution of Amino Acid Derived Vinyl Isocyanide Biosynthesis: Detection of an Elusive Intermediate. *Org. Lett.*, **2017**, *19*, 1208-1211.

44. Zhu, J.;G.M. Lippa;A.M. Gulick, and P.A. Tipton, Examining Reaction Specificity in PvcB, a Source of Diversity in Isonitrile-Containing Natural Products. *Biochemistry*, **2015**, *54*, 2659-2669.

45. Sio Charles, F.;G. Otten Linda;H. Cool Robbert, et al., Quorum Quenching by an N-Acyl-Homoserine Lactone Acylase from Pseudomonas aeruginosa PAO1. *Infect. Immun.*, **2006**, *74*, 1673-1682.

46. Denman, R.F.;D.S. Hoare, and E. Work, Diaminopimelic acid decarboxylase in pyridoxin-deficient Escherichia coli. *Biochim. Biophys. Acta*, **1955**, *16*, 442-443.

47. Nakano, Y. and S. Kitaoka, L-Aspartate α-Decarboxylase in a Cell-free System from Escherichia coli. *The Journal of Biochemistry*, **1971**, *70*, 327-334.

48. Ikai, H. and S. Yamamoto, Identification and analysis of a gene encoding L-2,4-diaminobutyrate:2-ketoglutarate 4-aminotransferase involved in the 1,3-diaminopropane production pathway in Acinetobacter baumannii. *J. Bacteriol.*, **1997**, *179*, 5118-5125.

49. Cheung, J.;F.C. Beasley;S. Liu, et al., Molecular characterization of staphyloferrin B biosynthesis in Staphylococcus aureus. *Mol. Microbiol.*, **2009**, *74*, 594-608.

50. Lyons, N.S.;A.N. Bogner;J.J. Tanner, and P. Sobrado, Kinetic and Structural Characterization of a Flavin-Dependent Putrescine N-Hydroxylase from Acinetobacter baumannii. *Biochemistry*, **2022**, *61*, 2607-2620.

51. Cotton, J.L.;J. Tao, and C.J. Balibar, Identification and Characterization of the Staphylococcus aureus Gene Cluster Coding for Staphyloferrin A. *Biochemistry*, **2009**, *48*, 1025-1035.

52. Shaw-Reid, C.A.;N.L. Kelleher;H.C. Losey, et al., Assembly line enzymology by multimodular nonribosomal peptide synthetases: the thioesterase domain of E. coli EntF catalyzes both elongation and cyclolactonization. *Chem. Biol.*, **1999**, *6*, 385-400.

53. Lin, H.;M.A. Fischbach;D.R. Liu, and C.T. Walsh, In Vitro Characterization of Salmochelin and Enterobactin Trilactone Hydrolases IroD, IroE, and Fes. *J. Am. Chem. Soc.*, **2005**, *127*, 11075-11084.

54. Perraud, Q.;L. Moynié;V. Gasser, et al., A Key Role for the Periplasmic PfeE Esterase in Iron Acquisition via the Siderophore Enterobactin in Pseudomonas aeruginosa. *ACS Chem. Biol.*, **2018**, *13*, 2603-2614.

55. Chao, J.D.;D. Wong, and Y. Av-Gay, Microbial Protein-tyrosine Kinases *. *J. Biol. Chem.*, **2014**, *289*, 9463-9472.

56. Speziali Craig, D.;E. Dale Suzanne;A. Henderson James, et al., Requirement of Staphylococcus aureus ATP-Binding Cassette-ATPase FhuC for Iron-Restricted Growth and Evidence that It Functions with More than One Iron Transporter. *J. Bacteriol.*, **2006**, *188*, 2048-2055.

57. Kolenbrander Paul, E.;N. Andersen Roxanna;A. Baker Rachel, and F. Jenkinson Howard, The Adhesion-Associated sca Operon

inStreptococcus gordonii Encodes an Inducible High-Affinity ABC Transporter for Mn2+ Uptake. *J. Bacteriol.*, **1998**, *180*, 290-295.
58. Friedrich, M.J.;L.C. de Veaux, and R.J. Kadner, Nucleotide sequence of the btuCED genes involved in vitamin B12 transport in Escherichia coli and homology with components of periplasmic-binding-protein-dependent transport systems. *J. Bacteriol.*, **1986**, *167*, 928-934.
59. Fischbach, M.A.;H. Lin;D.R. Liu, and C.T. Walsh, In vitro characterization of IroB, a pathogen-associated C-glycosyltransferase. *Proceedings of the National Academy of Sciences*, **2005**, *102*, 571-576.
60. Banerjee, S.;S. Paul;L.T. Nguyen, et al., FecB, a periplasmic ferric-citrate transporter from E. coli, can bind different forms of ferric-citrate as well as a wide variety of metal-free and metal-loaded tricarboxylic acids†. *Metallomics*, **2016**, *8*, 125-133.
61. Shea, C.M. and M.A. McIntosh, Nucleotide sequence and genetic organization of the ferric enterobactin transport system: homology to other peripiasmic binding protein-dependent systems in Escherichia coli. *Mol. Microbiol.*, **1991**, *5*, 1415-1428.
62. Miethke, M.;J. Hou, and M.A. Marahiel, The Siderophore-Interacting Protein YqjH Acts as a Ferric Reductase in Different Iron Assimilation Pathways of Escherichia coli. *Biochemistry*, **2011**, *50*, 10951-10964.
63. Zamboni, N.;E. Fischer;D. Laudert, et al., The Bacillus subtilis yqjI Gene Encodes the NADP+-Dependent 6-P-Gluconate Dehydrogenase in the Pentose Phosphate Pathway. *J. Bacteriol.*, **2004**, *186*, 4528-4534.
64. Eguchi, T.;Y. Dekishima;Y. Hamano, et al., A New Approach for the Investigation of Isoprenoid Biosynthesis Featuring Pathway Switching, Deuterium Hyperlabeling, and 1H NMR Spectroscopy. The Reaction Mechanism of a Novel Streptomyces Diterpene Cyclase. *The Journal of Organic Chemistry*, **2003**, *68*, 5433-5438.
65. Quatrini, R.;C. Appia-Ayme;Y. Denis, et al., Extending the models for iron and sulfur oxidation in the extreme Acidophile Acidithiobacillus ferrooxidans. *BMC Genomics*, **2009**, *10*, 394.
66. Welte, C.U.;R. de Graaf;P. Dalcin Martins, et al., A novel methoxydotrophic metabolism discovered in the hyperthermophilic archaeon Archaeoglobus fulgidus. *Environ. Microbiol.*, **2021**, *23*, 4017-4033.
67. Vepachedu Venkata, R. and G. Ferry James, Role of the Fused Corrinoid/Methyl Transfer Protein CmtA during CO-Dependent Growth of Methanosarcina acetivorans. *J. Bacteriol.*, **2012**, *194*, 4161-4168.
68. Wikström, M. and G. Hummer, Stoichiometry of proton translocation by respiratory complex I and its mechanistic implications. *Proceedings of the National Academy of Sciences*, **2012**, *109*, 4431-4436.
69. Dutta, T.K.;J. Chakraborty;M. Roy, et al., Cloning and characterization of a p-cymene monooxygenase from Pseudomonas chlororaphis subsp. aureofaciens. *Res. Microbiol.*, **2010**, *161*, 876-882.
70. Shaw, J.P. and S. Harayama, Purification and characterisation of the NADH: acceptor reductase component of xylene monooxygenase encoded by the TOL plasmid pWWO of Pseudomonas putida mt-2. *Eur. J. Biochem.*, **1992**, *209*, 51-61.
71. Yoshimi, A.;M. Tsuda, and C. Tanaka, Cloning and characterization of the histidine kinase gene Dic1 from Cochliobolus

heterostrophus that confers dicarboximide resistance and osmotic adaptation. *Mol. Genet. Genomics*, **2004**, *271*, 228-236.

72. Kowluru, A., Identification and characterization of a novel protein histidine kinase in the islet β cell: evidence for its regulation by mastoparan, an activator of G-proteins and insulin secretion. *Biochem. Pharmacol.*, **2002**, *63*, 2091-2100.

73. Kurth, J.M.;M.K. Nobu;H. Tamaki, et al., Methanogenic archaea use a bacteria-like methyltransferase system to demethoxylate aromatic compounds. *The ISME Journal*, **2021**, *15*, 3549-3565.

74. Pierce, E.;G. Xie;R.D. Barabote, et al., The complete genome sequence of Moorella thermoacetica (f. Clostridium thermoaceticum). *Environ. Microbiol.*, **2008**, *10*, 2550-2573.

75. Chen, H.;Z.K. Attieh;T. Su, et al., Hephaestin is a ferroxidase that maintains partial activity in sex-linked anemia mice. *Blood*, **2004**, *103*, 3933-3939.

76. Takai, M.;K. Kamimura, and T. Sugio, A new iron oxidase from a moderately thermophilic iron oxidizing bacterium strain TI-1. *Eur. J. Biochem.*, **2001**, *268*, 1653-1658.

77. Hudson, A.J.;S.C. Andrews;C. Hawkins, et al., Overproduction, purification and characterization of the Escherichia coli ferritin. *Eur. J. Biochem.*, **1993**, *218*, 985-995.

78. Stillman, T.J.;P.D. Hempstead;P.J. Artymiuk, et al., The high-resolution X-ray crystallographic structure of the ferritin (EcFtnA) of Escherichia coli; comparison with human H ferritin (HuHF) and the structures of the Fe3+ and Zn2+ derivatives11Edited by R. Huber. *J. Mol. Biol.*, **2001**, *307*, 587-603.

79. Bou-Abdallah, F.;H. Yang;A. Awomolo, et al., Functionality of the Three-Site Ferroxidase Center of Escherichia coli Bacterial Ferritin (EcFtnA). *Biochemistry*, **2014**, *53*, 483-495.
